# Susceptibility of broad reactivity nanobodies to resistance mutations in the S2 domain of SARS-CoV-2 predicted by yeast display deep mutational scanning

**DOI:** 10.3389/fimmu.2025.1726449

**Published:** 2026-01-12

**Authors:** Christina R. Ball, Walter Ramage, Ryan Mate, Simon E. Hufton

**Affiliations:** 1 Biotherapeutics and Advanced Therapies Division, Medicines and Healthcare products Regulatory Agency, Hertfordshire, United Kingdom; 2 Analytical and Biological Sciences, Medicines and Healthcare products Regulatory Agency, Hertfordshire, United Kingdom

**Keywords:** SARS-CoV-2, S2 domain, nanobody, yeast display, deep mutational scanning, pandemic

## Abstract

**Introduction:**

The rapid evolution of SARS-CoV-2 has led to the erosion of vaccine induced serum neutralization and monoclonal antibody efficacy. As such, interest is inevitably moving towards more conserved regions of the SARS-CoV-2 spike protein like the S2 domain. Resistance mutations continue to be a major obstacle for the development of antivirals and vaccines which target the RBD but what extent these will be a problem for S2 binding antibodies is not known.

**Methods:**

We have developed a yeast display deep scanning mutagenesis platform which allows an unbiased prospective assessment of millions of single and double mutations for their effects on antibody binding to the S2 domain.

**Results:**

We have compared the mutational resistance of a panel of five nanobodies mapped to four distinct non-competing epitopes within the conserved fusion peptide, stem helix and heptad repeat 2 elements of the S2 domain. Yeast display deep mutational scanning predicted reduced binding of C303, G223, G225, and G142 to naturally occurring resistance mutations which were experimentally confirmed on SARS-CoV-2 variants.

**Discussion:**

Our study shows that resistance mutations in conserved elements of the S2 domain may still pose a challenge to the development of monoclonal antibodies and subunit vaccines.

## 1 Introduction

Coronaviruses are zoonotic pathogens that in the last 20 years have been responsible for several epidemics and a pandemic, the former caused by severe acute respiratory syndrome coronavirus 1 (SARS-CoV-1), and the middle east respiratory syndrome coronavirus (MERS CoV) (1) and the latter by SARS-CoV-2. Coronavirus infection is a multistep process involving cleavage and rearrangement of the surface spike protein (2). The spike protein promotes viral entry through the interaction of the receptor binding domain (RBD) of the S1 subunit with its receptor on the host cell surface. Following binding, the more conserved S2 domain goes through structural rearrangements to mediate membrane fusion and release of viral RNA into the cell (1, 2). The patient’s immune response is mainly targeted towards the RBD domain (1) and its rapid evolution has led to the emergence of new variants which have consequently led to erosion of vaccine efficacy (3–6). In addition, all of the currently available therapeutic monoclonal antibodies (mAbs) target the RBD domain and have now either lost or have significantly reduced efficacy against these new variants (3, 4, 7). Sotrovimab™ is the only remaining therapeutic antibody that has retained some activity but could be expected to follow the same fate due to also binding to epitopes on the RBD (8). This disappointing response of the therapeutic monoclonal antibody field is in part due to initial efforts being focused on those mAbs with the highest neutralizing potency with less consideration given to antibodies to more conserved epitopes with lower potency in *in vitro* assays and perhaps different mechanisms of action. As with influenza hemagglutinin (HA) (9, 10) and HIV gp120 (11, 12), SARS-CoV-2 has parts of the spike protein which it cannot easily change without affecting virus function and these vulnerable parts of the spike protein can be targeted with antibodies. Analogous to the influenza HA stem region (13, 14), the S2 domain is required for the subsequent stages of infection and contains the membrane fusion machinery. This region is highly conserved and goes through complex structural rearrangements after viral attachment to cells. Post COVID-19 pandemic has seen a shift in focus away from evolutionarily susceptible regions to isolating monoclonal antibodies to more conserved epitopes like the S2 domain with the expectation that these will have broader reactivity (15–20). Nevertheless, choosing which monoclonal antibodies to develop to a constantly evolving target like the SARS-CoV-2 spike protein is a considerable challenge and the COVID-19 pandemic has highlighted that greater consideration should be given to predicting mutational escape and to focus on developing those monoclonal antibody therapeutics least susceptible to viral evolution. Targeting more conserved epitopes in the S2 domain is undoubtedly a promising option but as the S2 domain has shown evidence of antigenic shift in the core of the fusion machinery (21, 22), choosing antibodies which target this region still requires careful consideration.

Predicting which mutations pose a threat before they arise in patients is a significant challenge and generally limited to retrospective analysis (23). Historically, studying mutational escape has been carried out using live virus infection in the presence of mAbs and screening surviving virus (24, 25). Although widely used, this is low throughput and heavily biased particularly against those antibodies binding to functionally conserved epitopes that the virus cannot change easily without affecting viability. Indeed, many of the early mAbs binding to highly conserved epitopes on influenza HA stem were characterized by their resistance to escape even after using extensive virus passaging (9, 10, 24–26). In addition, using live viruses presents a potential risk of generating ‘gain of function’ mutations which could result in more lethal viruses resistant to the therapies being developed. High-throughput and unbiased approaches are needed capable of prospectively evaluating mutational escape from antibodies particularly to more conserved epitopes. This will facilitate development of more durable anti-viral mAbs with optimum resistance to escape. Since its inception, deep mutational scanning (DMS) has emerged as a powerful technology for engineering proteins including viral glycoproteins (27). By constructing comprehensive libraries of mutated viral proteins, it is now possible to search for escape mutations in parallel using *in vitro* selection technologies. Expression systems of libraries of influenza HA and SARS-CoV-2 have been described which use viruses (28), yeast display (29–32), or mammalian cell surface display (33) and each has their advantages and disadvantages. In all cases, libraries are incubated with antibodies before passaging through cell culture for virus-based technologies or selected for loss of antibody binding using fluorescence-activated cell sorting (FACS) for library display technologies.

Yeast display DMS combines gene mutagenesis with yeast display and next generation sequencing (29) to evaluate millions of mutations for their effect on binding and stability. We have previously displayed the complete influenza HA on yeast and used DMS to map nanobodies binding to the conserved stem region (29, 34). Given the structural similarities of the SARS-CoV-2 protein with influenza HA, we have tested if the complete spike protein and the S2 domain could be displayed. To date the size and complexity of the SARS-CoV-2 spike protein has presented challenges for yeast display DMS and has been limited to the RBD domain (30, 31). As the S2 domain is a current target of next generation monoclonal antibodies and subunit vaccines, it is important to assess susceptibilities to resistance mutations in functionally important elements like the fusion peptide (FP), stem helix (SH) and heptad repeat 2 (HR2) (35, 36). We have taken an unbiased approach to DMS and generated a random library of mutations displayed on yeast and showed that it can be used to survey the effects of both single and double mutations on antibody binding. We have further isolated a panel of nanobodies against conserved elements of the S2 domain and compared their mutational resistance. Their mutational escape profiles determined by yeast display DMS predicted resistance mutations, some of which correlated with circulating S2 mutations in naturally occurring variants. The identification of mutations to nanobodies binding highly conserved regions of the S2 domain indicates that escape mutations may still pose a challenge for S2 based vaccines and therapeutic monoclonal antibodies targeting conserved elements. Applications of yeast displayed S2 libraries to mitigate these risks and designing more durable monoclonal antibody therapeutics is discussed.

## 2 Materials and methods

### 2.1 Display of spike protein on the surface of yeast cells and flow cytometry

SARS-CoV-2 (Wuhan-Hu-1) S protein, S1 or S2 domains and RBD (Accession number QHD43416.1) genes were codon optimized for *S. cerevisiae* expression and synthesized (Integrated DNA Technologies, B.V. Belgium) as a gene or gblocks. S protein gene (V16-P1213), with proline substitutions; F817P, A892P, A899P, A942P, K986P, V987P and alanine substitutions (R683A and R685A) introduced to stabilize the prefusion state of the S protein and abolish the furin cleavage site respectively (37, 38) was synthesized as a gene, with *SfiI* and *NotI* restriction sites at the 5’ and 3’ ends respectively, and cloned into the pUCIDT-AMP plasmid. This plasmid was reconstituted in TE to 100 ng/µl as IDT instructions, 1 ng was used to transform JM109 *E. coli* (Promega, L2001) and plasmid prepared (Qiagen, 27106). 2.5 µg plasmid was digested with *SfiI* (New England Biolabs, R0123) at 50°C for 14 hours followed by *NotI* (New England Biolabs, R3189) at 37°C for 5 hour and the DNA purified using a PCR clean up kit (Qiagen, 28104). 100 ng *Sfii/NotI* digested S protein, with the pUCIDT-AMP plasmid still present, and 100 ng *SfiI/NotI* restricted and dephosphorylated (New England Biolabs, M0289S) pTQ6 (29) plasmid, were ligated (ThermoFisher Scientific, EL0013) for 1 hour at 22°C, the ligation reaction was heat inactivated for 10 min at 65°C and 2 µl used to transform NEB^®^ 5-alpha Competent *E. coli* (New England Biolabs, C2987H). Plasmid DNA was prepared from single colonies (Qiagen, 27106) and 100 ng sequenced S protein in pTQ6 used to transform *S. cerevisiae* EBY100 yeast cells (ThermoFisher Scientific, V835-01) using a yeast transformation kit (Sigma-Aldrich, YEAST1) as the manufacturer’s instructions. S1 domain (V16-R682), S2 domain (S686-P1213) with and without the proline substitutions as S protein above and RBD (R319-K537), all with overlapping sequences to allow homologous recombination cloning in yeast (29), were synthesized as gblocks. 100 ng *SfiI/NotI* restricted pTQ6 plasmid (29), and 100 ng of the synthesized gblocks (10 ng/µl in TE) were used to transform *S. cerevisiae* EBY100 yeast cells using the yeast transformation kit as above. Yeast clones containing the correct S protein gene sequence were stored at -70°C in 20% (v/v) glycerol. All yeast growth, induction of surface display and cell sorting was performed largely as in (29, 39). 2 ml of selection medium, SD/CAA (5 g/L Casamino acids (ThermoFisher Scientific, 223050), 7 g/L Yeast nitrogen base without amino acids (Sigma-Aldrich, Y0626), 10.19 g/L Na_2_HPO_4_·7H_2_O (Sigma-Aldrich, S9390), 8.56 g/L NaH_2_PO_4_·H_2_O (Sigma-Aldrich, S9638) with 20 g/L D-(+)-Glucose (Sigma-Aldrich, G8270), + 1x Penicillin (50 units/ml)-Streptomycin (50 µg/ml) (ThermoFisher scientific, P0781) was inoculated with a scrape of yeast from the frozen glycerol stock and shaken for ~28 hours at 30°C. To induce surface display of S protein, the growing yeast cultures were diluted into induction medium, SG/R + CAA (SD/CAA medium as above except with 1 g/L glucose (Sigma-Aldrich, G8270), plus 20 g/L D-(+)-Galactose (Sigma-Aldrich, 48260) and 20 g/L D-(+)-Raffinose (Sigma-Aldrich, R0250) + 1x Penicillin-Streptomycin) to give ~1 x 10^7^ cells/ml and shaken at 20°C for ~20 hours. After growth and induction, yeast cells were labelled in MultiScreen^®^ 96 well plates (Merck, MAGVS2210) for screening using flow cytometry. Approximately 2 x 10^5^ cells were mixed with 200 µl flow cytometry wash buffer (5 g/l bovine serum albumin (BSA) (Sigma-Aldrich, A7888), 2 mM ethylenediaminetetraacetic acid (EDTA) (avantor, 20302.260) in PBS) and pipetted into each well of the vacuum filter plate. The buffer was removed by vacuum filtration and the cells washed twice with 200 µl ice cold wash buffer. All labelling reagents were diluted in wash buffer, applied to the yeast cells and incubated for 1 hour (at room temperature with shaking for the first labelling step and at 4°C for subsequent staining steps). In between each labelling step, solution was removed by vacuum filtration, and the cells were washed twice with ice cold wash buffer. After the final staining step, cells were washed twice more and resuspended in 200 µl ice cold wash buffer then transferred to round bottomed 96 well plates (fisher scientific, 11313595). Yeast cells were separately labelled with 1:500 diluted (~2 µg/ml) mouse anti-V5-Tag (BIO-RAD, MCA1360) or 1 nM, or a concentration series, anti-RBD mAbs, CV30 (expressed and purified at MHRA) or Sotrovimab™ (AD Allen, Pharma) or S2 domain mAb, AS86 (Acro biosystems, S2N-S86). Display was detected using 1:500 diluted Alexa Fluor™ 488 goat anti-mouse IgG (H+L) (Invitrogen, A11029), human mAb binding was detected using 1:500 diluted Alexa Fluor™ 647-conjugated AffiniPure goat anti-human IgA + IgG + IgM (H+L) (Jackson ImmunoResearch, 109-605-064). For nanobody binding, yeast cells were co-labelled with 25 nM Nb, 1:500 diluted (~2 µg/ml) chicken anti c-Myc (Bethyl Laboratories, Inc, A190-103A) and mouse anti-V5-Tag as above to show S protein display. Display was detected as above and Nb binding using 1:500 diluted Alexa Fluor^®^ 647-conjugated AffiniPure™ Goat anti-chicken IgG (H+L) (Jackson ImmunoResearch, 103-605-155). Labelled yeast cells were analysed on a FACSCanto™ II flow cytometer (Becton Dickinson) and data analysed using BD Diva and FlowJo software.

### 2.2 Alpaca immunisation and nanobody library construction

To obtain cross-reactive SARS-CoV nanobodies, a juvenile male alpaca was obtained through the Royal Veterinary College, Hertfordshire, UK. and immunized by 6 intramuscular injections consisting of 100 µg of SARS-CoV-2 S protein trimer on days 0, 14, and 50 µg on day 29, 100 µg SARS-CoV-1 S protein trimer on days 43 and 62 then 100 µg of both on day 72. Both antigens used were recombinant proteins from Acro biosystems; SARS-CoV-2 S protein trimer (V16-P1213) (Wuhan-Hu-1, SPN-C52H8), and SARS S protein trimer (S14-P1195) (SPN-S52H5). Antigens were emulsified in 1 ml of TiterMax^®^ Gold Adjuvant (Signa-Aldrich, T2684) prior to injection in alternating legs. Preceding each injection, and 14 days after the final boost, blood was collected from which plasma was prepared and PBMNC’s isolated for RNA purification. All experiments were reviewed by a local ethics committee and performed under a UK Home Office License. Plasma was tested for binding to SARS-CoV-2 S protein trimer in an ELISA format using 1 µg/ml antigen (Wuhan-Hu-1, Acro biosystems, SPN-C52H8) and an anti-alpaca HRP conjugated secondary antibody (Jackson ImmunoResearch). Total RNA from the PBMNC’s was extracted (RNeasy^®^ mini kit, Qiagen, 74104) and transcribed into cDNA using the oligo-dT primer (SuperScript™ III First-Strand Synthesis System, ThermoFisher Scientific, 18080-051) following the manufacturer’s instructions. Two rounds of PCR were performed to specifically isolate alpaca Nb DNA which was cloned into a phagemid plasmid, pNIBS-1 (40). and transformed into TG1 cells (Agilent, 200123). The TG1 Nb library was infected with M13KO7 helper phage (New England Biolabs, N0315S) to present the Nb on the surface of phage for selection on antigen.

### 2.3 Selection and screening for cross-reactive S2 domain specific nanobodies

Phage antibody library selections to isolate Nbs that bind immobilized antigen were performed on the library essentially as in (40). All coating antigens were recombinant proteins from Acro biosystems. Phage displaying immobilized antigen specific Nbs, selection strategies (i) and (ii), were enriched by bio-panning on 12 μg antigen coated on Maxisorp Startubes (ThermoFisher Scientific, 470319) with a buffer only negative control for each panning round. Strategy (i) was alternating selections on immobilized full trimeric SARS-CoV-2 S protein (V16-P1213) (Wuhan-Hu-1, SPN-C52H8) followed by SARS-CoV-1 full trimeric S protein (S14-P1195) (SPN-S52H5), (Nbs labelled with a ‘C’ prefix) and strategy (ii) was two rounds of selection on immobilized SARS-CoV-2 S2 domain (S686-P1213) (Wuhan-Hu-1, S2N-S52H5), (Nbs labelled with an ‘F1’ or ‘F2’ prefix for each selection round). To select Nbs that bind S2 in solution, selection strategy (iii), the unselected library of Nb-displaying phage were blocked in 2% (w/v) Marvel™ milk powder in phosphate-buffered saline (MPBS) then incubated with 12.5 µg biotinylated SARS-CoV-2 S2 (S686-P1213) (Wuhan-Hu-1, S2N-S52E8), for 1 hour at room temperature with rolling. To isolate the Nb displaying phage that bound to biotinylated S2, 200 µl blocked and washed Dynabeads™ M-280 Streptavidin (ThermoFisher Scientific, 11205D) were added to the unselected phage Nb library/biotinylated S2 mixture and incubated for 15 min at room temperature on a rolling platform. Beads were washed three times with MPBS, three times in PBS + 0.1% (v/v) Tween 20 and three times with PBS. Washed beads were reconstituted in 120 µl PBS and 100 µl mixed with 4.9 ml of 2 x YT plus 5 ml *Escherichia coli* ER2738 (New England Biolabs, E4104) culture grown to an OD _600nm_ of 0.5. Following incubation in a water bath at 37°C for 30 minutes, the 10 ml culture was centrifuged and the cell pellet reconstituted in 2 ml 2 x YT and spread onto 24 cm bioassay dishes (ThermoFisher Scientific, 166508) containing 2 x YT agar supplemented with 100 µg/ml (w/v) carbenicillin and 2% (w/v) glucose. Plates were grown overnight at 37°C and harvested. This S2 selected Nb displaying phage library was infected with M13KO7 helper phage (New England Biolabs, N0315S) and a second round of bio-panning performed as above. The recovered Nbs from these selections were labelled with a ‘G1’ or ‘G2’ prefix for round 1 or 2 respectively. Phage titres before and after selection were determined for all panning rounds. Primary screening was carried out using soluble Nbs harvested from induced culture supernatant in a 96 well format. For Nbs selected on immobilized antigen, Maxisorp 96-well plates (ThermoFisher Scientific, 442404) were coated with 100 μl antigen at 1.2 μg/ml in PBS overnight at 4°C. All coating antigens were recombinant SARS-CoV-2 (Wuhan-Hu-1) proteins from Acro biosystems; S protein (V16-P1213) (SPN-C52H8), RBD (R319-K537) (SPD-C52H3), S2 (S686-P1213) (S2N-C52H5). Plates were blocked with 120 μl/well MPBS. For Nbs selected on S2 in solution, Streptavidin Coated Clear 96-well Plates with SuperBlock™ Blocking Buffer (ThermoFisher Scientific, 15124) were incubated with 100 µl of biotinylated S2 (S686-P1213) (S2N-S52E8), at 1 µg/ml in MPBS for 2 hours in a shaking incubator. After coating, induced culture supernatant in 2% marvel (w/v) was added to all plates and incubated 1.5 hour in a shaking incubator. To detect Nb binding, 100 μl of anti c-Myc-peroxidase clone 9E10 (Roche Diagnostics GmbH, 11667149001) at 1:1000 dilution in MPBS was added for 1 hour at room temperature and developed using TMB (3,3’,5,5’ tetramethylbenzidine) and detection at OD _450nm_ on a VERSAmax microplate reader (Molecular Devices).

### 2.4 Expression and purification of nanobodies

For large scale expression, monovalent Nbs were transformed into BL21 (New England Biolabs, C2350H) as manufacturer’s instructions then expression and purification was as previously described (40). All Nbs were fused to a C-terminal c-Myc tag for detection in either FACS or ELISA. S2 binding Nbs; C303, F226, G223, G225, and G142, were reformatted as human IgG1 Fc fusions (Nb-hIgG1 Fc), transiently expressed from XtenCHO cells for seven days, Protein A purified and dialyzed into PBS pH 7.5 (ProteoGenix).

### 2.5 Enzyme-linked immunosorbent assay

To test binding of monovalent Nbs to immobilized antigen, Maxisorp 96-well plates (ThermoFisher Scientific, 442404) were coated with 100 μl antigen at 0.75 μg/ml in PBS overnight at 4°C. All antigens used were recombinant proteins from Acro biosystems; SARS-CoV-2 RBD (Wuhan-Hu-1, SPD-C52H3), SARS-CoV-2 RBD (Omicron variant/B.1.1.529, SPD-C522e) SARS-CoV-2 S proteins, (Wuhan-Hu-1, SPN-C52H9; Delta variant/B.1.617.2, SPN-C52He; Omicron variant/B.1.1.529, SPN-C52Hz); SARS S protein (CoV-1, SPN-S52H6); Pangolin coronavirus hCoV-19/pangolin/Guangdong/1/2019 S protein (R677A, KV978-979AA) (SPN-P52H3); Bat coronavirus HKU3 (BtCoV) S protein (R654A, KV955-956PP), (SPN-B52H7); SARS-CoV BtKY72 S protein (K669A, KV971-972PP) (SPN-S52Hu); HCoV-OC43 S protein (SPN-H52Hz); HCoV-NL63 S protein (SPN-H52H4); MERS S protein (R748A, R751A, V1060P, L1061P) (SPN-M52H5); SARS-CoV-2 S2 domains (Wuhan-Hu-1, S2N-C52H5 and Omicron variant/BA.2, S2N-C52Hh). Plates were blocked with 120 μl/well MPBS prior to Nbs being added at 10 µg/ml (100 µl) or for the binding curves, 100 μl of serial dilutions of Nb in MPBS for 2 hours at room temperature. For monovalent Nbs selected on biotinylated S2 in solution, Streptavidin Coated Clear 96-well Plates with SuperBlock™ Blocking Buffer (ThermoFisher Scientific, 15124) were incubated with 100 µl of biotinylated S2 at 0.75 µg/ml in MPBS for 2 hours in a shaking incubator. Antigens used were Biotinylated SARS-CoV-2 S2 (Wuhan-Hu-1, S2N-C52E8) or (Omicron variant/BA.2, S2N-C82E4). Nbs were added at 10 µg/ml (100 µl) or for the binding curves, 100 μl of serial dilutions of Nb in MPBS were added and incubated for 2 hours at room temperature. To detect Nb binding, 100 μl of anti c-Myc-Monoclonal Antibody (9E10), HRP (ThermoFisher Scientific, MA1-81357) at 1:1000 dilution in MPBS was added for 1 hour at room temperature and developed using TMB and detection at OD _450nm_ on a VERSAmax microplate reader (Molecular Devices). The data was plotted and EC50’s determined in GraphPad Prism 10 software. To test binding of Nb-hIgG1 Fc, Streptavidin Coated Clear 96-well Plates with SuperBlock™ Blocking Buffer (ThermoFisher Scientific, 15124) were incubated with 100 µl of biotinylated antigen at 0.5 µg/ml in MPBS for 2 hours in a shaking incubator. Antigens used were all recombinant proteins from Acro biosystems; Biotinylated SARS-CoV-2 S proteins (Wuhan-Hu-1, SPN-C82E9; Delta variant/B.1.617.2, SPN-C82Ec; Omicron variant/BA.2, SPN-C82Er; Omicron variant/BA.2.75.2, SPN-C82Ex; Omicron variant/BA.2.86, SPN-C82Q1); Biotinylated SARS S protein (CoV-1, SPN-S82E3); Biotinylated MERS S protein (SPN-M82E3). 100 μl of serial dilutions of Nb-hIgG1 Fc in MPBS were added and incubated for 2 hours at room temperature. To detect Nb-hIgG1 Fc binding, 100 μl of goat anti human IgG (Fc specific) - HRP (Sigma-Aldrich, A0170) at 1:1000 dilution in MPBS was added for 1 hour at room temperature and developed using TMB and detection at OD _450nm_ on a VERSAmax microplate reader (Molecular Devices). The data was plotted and EC50’s determined in GraphPad Prism 10 software.

### 2.6 Epitope binning and affinity determination of nanobodies using surface plasmon resonance

Epitope binning was done on a BIAcore T100 machine (T200 sensitivity enhanced) (GE healthcare) on Series S Sensor Chip SA (Cytiva, 29104992) streptavidin chips. Chips were loaded with biotinylated S2 (S686-P1213) (Wuhan-Hu-1, Accession No. QHD43416.1, Acro biosystems, S2N-S52E8), according to manufacturer’s instructions using manufacturer supplied reagents. Purified nanobody 1 was injected over the S2 loaded surface at 250 nM for 300 seconds to saturate the surface followed by injection of nanobody 2 at 100 nM for 200 seconds. Sensograms were analysed using BIAevaluation 3.1 software and an increase in resonance units following injection of the second nanobody indicated that the two nanobodies being tested bound to non-competing epitopes. If there was no substantial increase in resonance units, the nanobodies were seen as recognizing overlapping epitopes. The assay was performed on nanobodies C303, F226, G223, G225, and G142 with each nanobody being tested against the other as either nanobody 1 or nanobody 2. Affinity was determined using single cycle kinetics with biotinylated SARS-CoV-2 S2 and full trimeric spike proteins immobilized on a Series S sensor chip SA streptavidin chip as above. All biotinylated antigens were purchased from Acro biosystems and were the same as those used in ELISA. A concentration series of purified Nbs was flowed over the surface and sensograms analysed by BIAevaluation software 3.1.

### 2.7 Construction of a random mutagenised SARS-CoV-2 S2 domain library

A library of S2 mutants was generated by error-prone PCR using oligonucleotides Fw_YD.pTQ6_SfiI (5’ – TGGTGGCGGAGGTTCTGCGGCCCAGCCGGCC – 3’) and Rev_YDpTQ6_NotI (5’ – GGTTTGGGATTGGCTTACCA – 3’) using the GeneMorph II Random Mutagenesis Kit (Agilent Technologies, 200550) according to the manufacturer’s instructions for a low mutation frequency (640 ng S2 gene template). PCR; 1x 95°C – 2 min, 25x (95°C – 30s, 64°C – 30s, 72°C – 100s), 72°C – 10 min. 14 µg of error-prone PCR product was co-transfected with 14 µg of *SfiI/NotI* digested pTQ6 plasmid into EBY100 competent cells using a yeast transformation kit (Sigma-Aldrich, YEAST1) as manufacturer’s instructions and the library amplified as in (29). The final library size was determined through serial dilutions on selective plates.

### 2.8 Fluorescence-activated cell sorting of the randomly mutated S2 library

For cell sorting, 40 ml SD/CAA was inoculated with ten times the size of the randomly mutated S2 library, and incubated at 30°C, with shaking for ~ 17 hour. To induce S2 display on the yeast surface, 1.7 x 10^8^ cells were added to 10 ml SG/R + CAA and incubated at 20°C, with shaking for ~ 20 hour. 4 x 10^7^ cells were pelleted, washed and labelled in 1.5 ml sterile microcentrifuge tubes using 1 ml volumes. All five Nbs were used at 200 nM for staining with all other labelling reagents being used at the same concentrations as previously. Flow cytometric cell sorting was performed on a FACSAria™ III (Becton Dickinson) cell sorter. Cells that display S2 (detected by the V5 tag) but show an absence of binding to each Nb (detected by the c-Myc tag on the Nb), lower right quadrant of the FACS dot plot, were sorted into 2 ml SD/CAA and shaken at 30°C for ~17 hours. Each culture was diluted to 20 ml, incubated for a further 24 hours, then cells pelleted and resuspended in 5ml SD/CAA. Aliquots in 20% final glycerol were stored at -80°C. A second round of sorting was performed on each Nb sorted library using a non-competing Nb with the positive populations (upper right quadrant) collected. Plasmid DNA from the unselected and all selected libraries was extracted using the Zymoprep™ Yeast Plasmid Miniprep II kit (Zymoresearch, D2004) as manufacturer’s instructions.

### 2.9 Next generation sequencing of unselected and selected yeast displayed libraries

For next generation sequencing (NGS), S2 was PCR amplified from 50–100 ng plasmid DNA from the unselected and all Nb sorted yeast libraries in four ~500 bp fragments using primers that include both gene-specific (upper case) and Illumina adapter (lower case) sequences:

Fragment 1;
pTQ6_NGS_Fwd
5’ – tcgtcggcagcgtcagatgtgtataagagacagGTATGTTTTTGGAGGCGGAGGTTCTG – 3’
S2_NGS_Rev 1
5’ – gtctcgtgggctcggagatgtgtataagagacagCCAGCATCCGCCAGGGTAACC – 3’
Fragment 2;
S2_NGS_Fwd 2
5’ – tcgtcggcagcgtcagatgtgtataagagacagGTGGCTTCAATTTTTCACAGATCTTGC – 3’ S2_NGS_Rev 2
(5’ – gtctcgtgggctcggagatgtgtataagagacagCTTAACAAGTGTATTTAGGGCTTGTGC – 3’
Fragment 3
S2_NGS_Fwd 3
5’ – tcgtcggcagcgtcagatgtgtataagagacagCGCCATCGGTAAAATACAAGATTCC – 3’
S2_NGS_Rev 3
5’ – gtctcgtgggctcggagatgtgtataagagacagCTTTCCGTCATGACAAATTGCTG – 3’
Fragment 4
S2_NGS_Fwd 4
5’ – tcgtcggcagcgtcagatgtgtataagagacagCTGATGAGCTTTCCTCAATCCGCCC – 3’
S2_NGS_Rev 4
5’ – gtctcgtgggctcggagatgtgtataagagacagCAGATCAGCGGGTTTAAACGATAACAGTG – 3’

Amplification was performed using Platinum™ SuperFi™ II DNA Polymerase (ThermoFisher Scientific, 12361010) PCR; 1x 98°C – 30s, 25x (98°C – 10s, 60°C – 10s, 72°C – 20s), 1x 72°C – 5 min. The products were purified using AMPure XP beads (Beckman Coulter, A63881) at 1x ratio using manufacturer’s instructions and used as templates for index PCR using KAPA HiFi HotStart ReadyMix (Roche, 07958935001) and IDT for Illumina DNA/RNA UD Index set A, Tagmentation (Illumina, 20027213) using 7 cycles; 1x 95°C – 3 mins, 7x (95°C – 30s, 55°C – 30s, 72°C – 30s), 1x 72°C – 5 mins. Library amplicons were purified using AMPure XP beads (Beckman Coulter, A63881) as above. Quantification was performed on each library using Quant-iT™ 1X dsDNA Assay Kit, high sensitivity (ThermoFisher Scientific, Q33232). Amplicon libraries were pooled equimolarly and quantified using Qubit™ 1X dsDNA High Sensitivity Assay kit (ThermoFisher Scientific, Q33231). Quality check was performed using the Agilent DNA 1000 Kit for 21000 Bioanalyzer systems (Agilent, 5067-1504). The pooled library was sequenced on an Illumina NextSeq 2000 platform (Illumina) at 300 bp paired end reads.

### 2.10 Sequence analysis and bioinformatics

Adapter and quality trimming were performed using Cutadapt (v3.4) (Q20, minimum length 297 bp). For each amplicon, trimmed files were transferred to Geneious Prime (v2020.0.3.) where forward and reverse reads were paired, merged using BBMerge (‘normal’ setting) and translated into the correct reading frame. Further sequence editing was performed in Microsoft^®^ Excel^®^ (v2404 Build 16.0.17531.20190) to obtain a sequence list containing only open reading frame sequences of the desired amplicon length. To extract the single mutant population for amino acid positional counting a custom Excel formula was used which compared the amino acid differences between each sequence and the wild-type. A count of each of the 20 amino acids at each position in the amplicon was calculated.

(a) = count for each of the 20 different amino acids
(r) = position in the S2 sequence

To mitigate the effect of zero values a pseudocount of 1 was added to all counts. A count of 20 was therefore added to each total count of single mutants and the percentage frequency of every amino acid at each position calculated.


$$\text{Amino acid frequency }\left(\%\right)\;=\frac{\left(\text{Count of }\left(\text{a}\right)\text{ at position }\left(\text{r}\right)\right)}{\left(\text{Total count of single mutants}\right)}\text{x }100$$

To generate a positional mutational scan, the cumulative frequency of all mutations at each position (r) in the S2 protein was calculated.


$$\text{Positional enrichment}=\frac{\text{Cumulative Mutant Frequency at position }\left(\text{r}\right)\text{ post}-\text{selection}}{\text{Cumulative Mutant Frequency at position }\left(\text{r}\right)\text{ pre}-\text{selection}}$$

Positional enrichment was then plotted against each position in the S2 protein to generate a mutational scan.

To generate heatmaps showing the effect of each individual amino acid mutation in the S2 protein on binding of the specific nanobodies, the escape fraction of each individual amino acid (a) mutation at position (r) was calculated:


$$\text{Escape Fraction }=\frac{\text{Mutant frequency }\left(\%\right)\text{ of }\left(\text{a}\right)\text{ at position }\left(\text{r}\right)\text{ post}-\text{selection}}{\text{Mutant frequency }\left(\%\right)\text{ of }\left(\text{a}\right)\text{ at position }\left(\text{r}\right)\text{ pre}-\text{selection}}$$

The Log2 transformation of the escape fraction was plotted in GraphPad Prism.

### 2.11 Construction of specific S2 mutants

Single point mutations in S2 shown to have escaped binding to Nb, or the naturally occurring mutations to date, were introduced into Wuhan-Hu-1 S2 by site-directed mutagenesis using a QuickChange II Site Directed Mutagenesis kit (Agilent, 200524-5) as manufacturer’s instructions and cloned into EBY100 yeast using the yeast transformation kit as previously. Yeast clones were separately labelled for S2 display and Nb binding, and the extent of binding relative to S2 display analysed by flow cytometry.

## 3 Results

### 3.1 Display of SARS-CoV-2 spike on the surface of yeast

**Figure 1:**
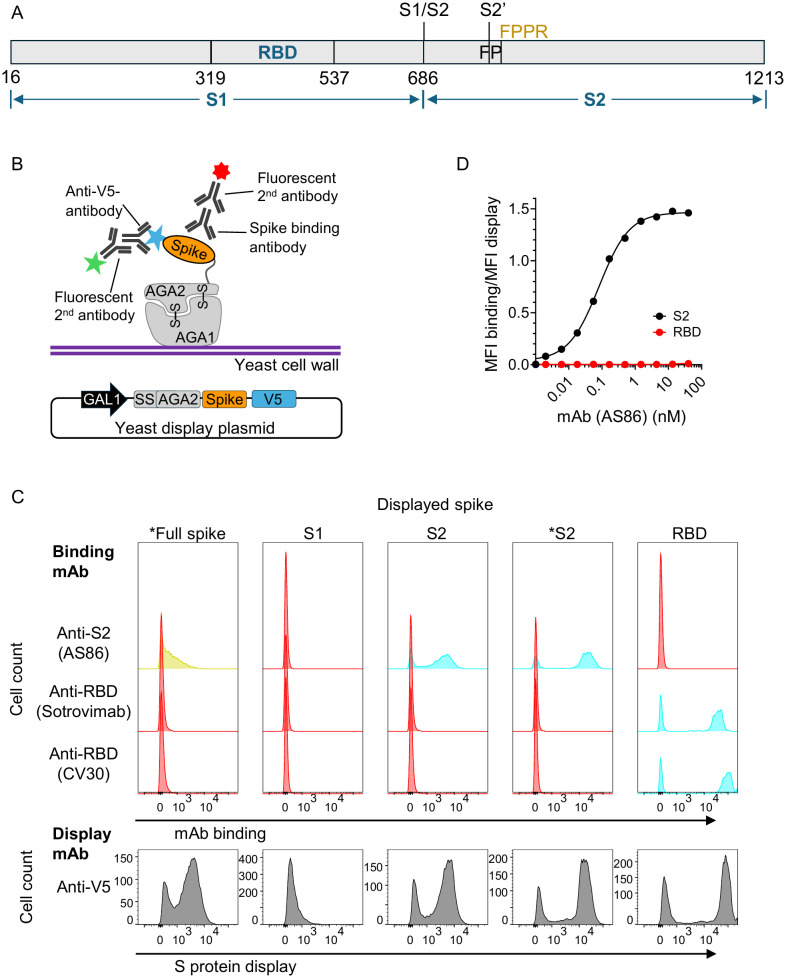
Display of full spike protein and sub-domains on yeast. **(A)** Linear representation of the SARS-CoV-2 spike protein showing the S1, RBD, and S2 domains. RBD, receptor binding domain; S1/S2, furin cleavage site; S2’, TMPRSS2 or cathepsin cleavage site; FP, fusion peptide; FPPR, fusion peptide proximal region. **(B)** Schematic of the yeast display plasmid and cell surface display of spike protein and detection with labelling antibodies. **(C)** Histograms showing spike protein display (grey plots) and binding (cyan), no binding (red) or intermediate binding (yellow) of human anti-S2 (AS86) or anti-RBD mAbs (Sotrovimab or CV30) by FACS. Display of Wuhan full spike or S2 domain with stabilizing prolines and the furin cleavage site, RRAR, mutated to RAAA (37, 38) are marked with an asterisk. **(D)** Plot of Mean fluorescence intensity (MFI) for binding divided by MFI for display for the human anti-S2 mAb (AS86) on Wuhan-Hu-1 S2 or RBD displayed on yeast.

To assess if domains other than the RBD domain could be displayed on yeast we cloned; (i) full spike (residues V16-P1213) with stabilizing proline mutations and abolished furin cleavage site (37, 38) (ii) wild-type S1 domain (residues V16-R682) (iii) S2 domain without stabilizing proline mutations (residues S686-P1213) (iv) S2 domain containing stabilizing proline mutations and (v) RBD domain (R319-K537) (**Figure 1A**) into a yeast display vector (29). Each spike protein/domain was fused at its N-terminus to the AGA2 yeast cell surface anchor protein and a C-terminal V5 tag (**Figure 1B**). Display and correct folding was tested by co-staining yeast cells with an anti-V5 mAb to show display (grey plots), and human anti-S2 or anti-RBD mAbs to show binding (cyan, yellow or red plots) by flow cytometry. The full spike protein, S2 domain, with and without the stabilizing proline mutations, and the RBD could be successfully displayed on the yeast cells surface (**Figure 1C**). The S1 domain, in our hands, was not displayed on yeast. Both S2 constructs, and to a lesser extent, the full spike bound a human S2 specific monoclonal antibody (AS86) isolated from a SARS-CoV-2 infected patient (**Figures 1C, D**). The RBD specific mAbs Sotrovimab™ and CV30 only bound RBD but not full spike or the S2 domain. As AS86 binds to the S2 domain in a functional ELISA, we reasoned that the S2 domain was displayed on the yeast cell surface in a correct conformation and could be used for deep mutational scanning.

### 3.2 Construction of a random mutagenised SARS-CoV-2 S2 spike domain library

Significant effort has focused on identifying and predicting individual mutations in SARS-CoV-2, however, combinations of mutations that have synergistic effects are less well studied despite having a significant role to play in viral evolution (41, 42). Random mutagenesis using error-prone PCR is a quick and economical way to generate both single mutations and random combinations of mutations. We constructed a random S2 library of 3.8 x 10^6^ clones using error-prone PCR (29) and library diversity was assessed by next generation sequencing of the unselected library using four overlapping amplicons spanning the entire 528 amino acid S2 gene (**Supplementary Figure S1A**). Sequencing reads of each amplicon were processed and the population of genes carrying single and double mutations were extracted as separate populations for analysis (**Supplementary Figure S1B**). After extraction of the single mutant population, we estimated the frequency of each amino acid across the S2 gene. This analysis showed that all positions in the S2 gene had been sampled with between 3–12 different mutations at each position with no evidence of any mutation being over-represented in the library (**Supplementary Figure S1C**). The total single mutant library diversity across the full S2 gene was 4445 (43%) out of a total theoretical maximum diversity of 10,560 single amino acid mutations. The remainder of the library contained S2 genes with combinations of mutations (**Supplementary Figure S1B**).

### 3.3 Isolation of broad reactivity nanobodies against sarbecovirus

**Figure 2:**
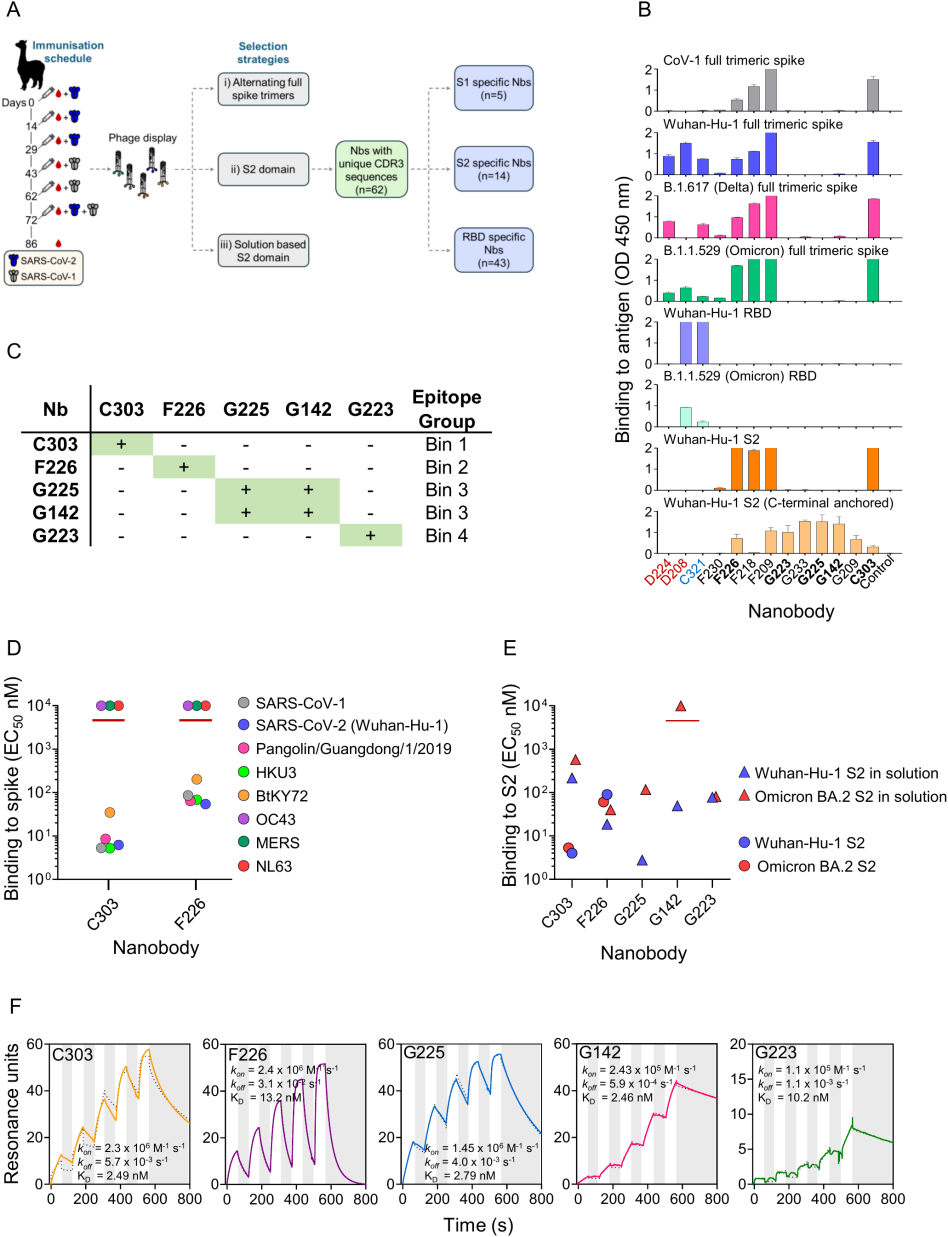
Isolation and Characterisation of broad reactivity nanobodies against the S2 domain of SARS-CoV-2. **(A)** Alpaca immunisation schedule and selection strategies to isolate broad reactivity Nbs against the S2 domain of SARS-CoV-2 (**Supplementary Figure S2A**). **(B)** ELISA of nanobodies on SARS-CoV-2 variants Wuhan-Hu-1 through to B.1.1.529 (Omicron). Specificity against SARS-CoV-1 and the S2 domain is also shown. Anti-S2 Nbs are labelled in black, example S1 specific Nbs in red and an RBD specific Nb in blue. Nbs in bold were taken forward for deep mutational scanning. An anti-H1 HA Nb is included as negative control. **(C)** Competition SPR of the five chosen Nbs, (+) and green box indicates competition whereas a (-) indicates no competition (**Supplementary Figure S2C**) **(D)** Binding (EC_50_ in nM) of anti-S2 Nbs F226 and C303 against immobilized betacoronavirus genus full trimeric spikes. Values above the red line indicate no binding. **(E)** Binding (EC_50_ in nM) of anti-S2 Nbs to SARS-CoV-2 strains Wuhan-Hu-1 or Omicron BA.2 S2 domains immobilized via a C-terminal biotin tag (triangles) or directly on an ELISA plate (circles). The value above the red line indicates no binding. **(F)** Affinity determination using single cycle kinetics on biotinylated Wuhan-Hu-1 S2. White and grey stripes indicate association and dissociation phases respectively. Sensograms are shown as full colour lines with dotted lines representing a 1:1 fitting model.

To bias for a broad reactive immune response we immunized an alpaca with SARS-CoV-2 and boosted with SARS-CoV-1 trimeric spike carrying proline and alanine substitutions to lock the antigen in the prefusion conformation (37, 38) (**Figure 2A**, **Supplementary Figure S2A**). Peripheral blood mononuclear cells were isolated and used to construct a nanobody (Nb) phage display library of size 4.3 x 10^8^ independent clones. To isolate broad reactive Nbs, three selection campaigns were carried out; (i) selection on immobilized SARS-CoV-2 followed by SARS-CoV-1 full trimeric spikes, (Nbs labelled with a ‘C’ prefix) (ii) two rounds of selection on immobilized Wuhan-Hu-1 S2 domain, (Nbs labelled with an ‘F’ prefix) (iii) two rounds of solution based selection on biotinylated Wuhan-Hu-1 S2 domain, (Nbs labelled with a ‘G’ prefix) (**Figure 2A**). We isolated a total of 62 nanobodies in primary screening with unique CDR3 sequences which were grouped as S1 domain specific (*n= 5*), RBD specific (*n=43*) and S2 domain specific (*n=14*). Nbs were then assessed for broad reactivity to SARS-CoV-2 variants that had emerged during the COVID-19 pandemic and the divergent sarbecovirus SARS-CoV-1 (**Figure 2B**). Competition surface plasmon resonance (SPR) placed the anti-S2 Nbs into four epitope bins. Five Nbs, with the broadest reactivity and unique CDR3 sequences; C303, F226, G225, G142, and G223 were chosen for deep mutational scanning (**Figure 2C**, **Supplementary Figure S2C**). Although G225 and G142 had different CDR3 sequences and were both placed in epitope bin 3, we tested if deep mutational scanning could detect differences in their escape profiles. F226 and C303 bound the prefusion-stabilized trimeric spike of all clades of sarbecovirus but not OC43 (embecovirus), MERS (merbecovirus) or NL63 (alphacoronavirus) (**Figure 2D**, **Supplementary Figure S2B**) as well as the S2 domains of Wuhan-Hu-1 and Omicron BA.2 immobilized on an ELISA plate (**Figure 2E**, **Supplementary Figure S2D**). All five Nbs bound biotinylated Wuhan-Hu-1 S2 domain anchored via the C-terminal biotin tag in ELISA. All Nbs bound similarly anchored Omicron BA.2 in ELISA except for G142 which shows total loss of binding and G225, which shows >100-fold reduction in binding (**Figure 2E**, **Supplementary Figure S2D**). G223 and G225/G142, only bound to biotinylated S2 domain which was consistent with their being isolated from the solution-based selection (**Figure 2A**). SPR was used to determine the affinity of binding of these five Nbs to biotinylated Wuhan-Hu-1 S2 domain (**Figure 2F**). A summary of the properties of the S2 specific nanobodies is shown in **Table 1**. All five nanobodies were tested for neutralization as both monovalent nanobodies and as nanobody Fc fusions but in neither case were we able to detect any appreciable neutralization activity in a lentiviral pseudovirus system (43). The potency of S2 specific antibodies is well documented as being orders of magnitude lower than RBD specific antibodies in *in vitro* assays and does not necessarily reflect their potential efficacy *in vivo* (17, 18, 44–46). This inconsistency is not uncommon and may reflect differences in the geometry, density and presentation of spike protein during the cell entry step in pseudovirus compared to the *in vivo* situation or in fact an increased protective role for effector functions in non-neutralizing antibodies against SARS-CoV-2 (44).

**Table 1:** Binding properties of the S2 specific nanobodies.

Test	Antigen	Nanobody					
		C303	F226	G225	G142	G223	Control
Binding ELISA (EC_50_ nM)	Wuhan-Hu-1 RBD	–	–	–	–	–	+
	Omicron B.1.1.529 RBD	–	–	–	–	–	+/-
	Wuhan-Hu-1 S2	4.0	89.8	–	–	–	–
	Omicron BA.2 S2	5.3	60.7	NT	NT	NT	NT
	Biotinylated Wuhan-Hu-1 S2	216.7	18.6	2.76	49.2	77.9	–
	Biotinylated Omicron BA.2 S2	574.6	40.1	116.8	NB	81.0	–
	NL63	–	–	NT	NT	NT	–
	OC43	–	–	NT	NT	NT	–
	MERS	–	–	NT	NT	NT	–
	SARS-CoV-1	5.3	85.5	NT	NT	NT	–
	Wuhan-Hu-1	6.2	54.1	NT	NT	NT	+
	Omicron B.1.1.529	+	+	–	–	–	+/-
	Pangolin/Guangdong/1//2019	8.5	64.0	NT	NT	NT	+
	HKU3	5.2	67.9	NT	NT	NT	–
	BtKY72	34.7	201.3	NT	NT	NT	+
SPR Competition	Wuhan-Hu-1 S2	Bin 1	Bin 2	Bin 3	Bin 3	Bin 4	NT
SPR Affinity (nM)	Wuhan-Hu-1 S2	2.49	13.2	2.79	2.46	10.2	NT
	Omicron (BA.2) S2 (N764K)	1.5	21.8	283	$	39	NT
	SARS-CoV-1	3.3	24	84	$	$*	NT
	Wuhan-Hu-1	1.2	116	2.1	1.8	~0.1*	NT
	Delta (D950N)	1.26	117	2.6	1.4	$ *	NT
	Omicron (BA.2) (N764K)	1.6	141	137	$	~0.1*	NT
	Omicron BA.2.75.2 (N764K, D1199N)	$	62.8	77.5	$	0.22*	NT
	Omicron BA.2.86 (N764K, S939F)	1.28	51	$	$	$*	NT
	MERS	166	$	$	$	$*	NT
Flow cytometry	Wuhan-Hu-1 RBD	–	–	–	–	–	+
	Wuhan-Hu-1 S2	+	+	+	+	+	–

ELISA on immobilized antigens, except for biotinylated Wuhan-Hu-1 S2 and Omicron BA.2 S2 which were immobilized on a streptavidin plate. ‘+’ indicates binding and ‘–’ indicates no binding. EC_50_ values in nM. NT is not tested. Surface plasmon resonance (SPR) determination of Nb competition and affinity (k_D_) in nM against spike proteins SARS-CoV-1, MERS, SARS-CoV-2 Wuhan-Hu-1 and naturally occurring variants. ‘$’ indicates no binding or binding could not be fitted with a 1:1 model using BIAevaluation software.’*’ indicates binding was evaluated using G223-IgG1 Fc fusion. All other affinities were determined using purified monovalent Nb. Control is RBD binding nanobody C321. For both ELISA and SPR determinations, antigens shaded in grey are full trimeric spike. Specific Nb binding against the S2 domain displayed on the surface of yeast demonstrated in flow cytometry.

### 3.4 Yeast display deep mutational scanning of S2 specific nanobodies

**Figure 3:**
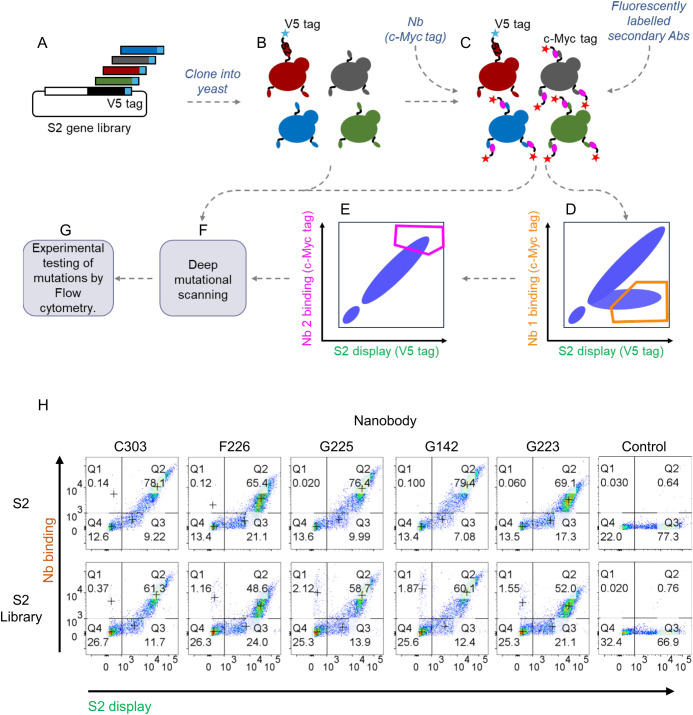
Yeast display deep mutational scanning. **(A)** Error-prone PCR followed by **(B)** yeast recombination cloning to generate a mutagenised S2 library displayed on the surface of yeast. **(C)** The library was co-stained with anti-V5 mAb (display), and anti-S2 Nbs C303, F226, G223, G225 or G142 plus anti-c-Myc mAb, (Nb binding). **(D)** The population of S2 mutations that escape binding (orange gate) were collected by fluorescence-activated cell sorting (FACS). **(E)** A second round of FACS, (folding reporter) using a non-competing Nb, was used to collect mutated S2 genes which were correctly folded (pink gate). **(F)** All output libraries were analysed using next generation sequencing and the escape fraction calculated. **(G)** Individual mutations identified by deep mutational scanning were validated by site directed mutagenesis of S2 and experimentally tested in flow cytometry. **(H)** Display and Nb binding to wild-type S2 or the unselected mutated S2 library by flow cytometry. Control is a nanobody (C321) against the RBD domain.

**Figure 4:**
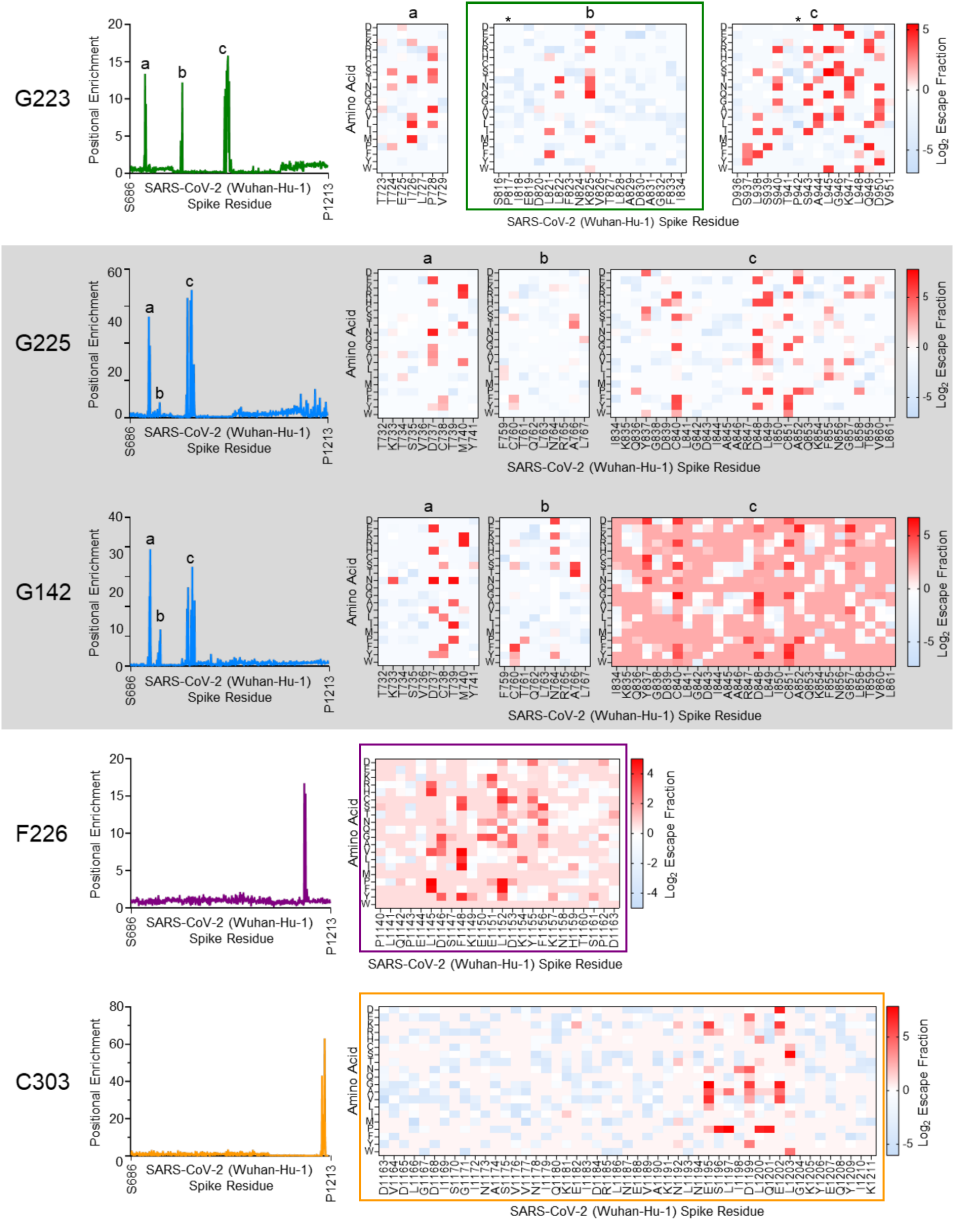
Mutational escape profiles of S2 specific nanobodies. Mutational scans show positional enrichment plotted against S2 residue S686 – P1213 are G223 (green), G225/G142 (blue), F226 (purple), C303 (yellow). Peaks represent S2 residue positions at which mutations interfere with nanobody binding. Heatmaps corresponding to these peaks indicate escape fractions for each possible amino acid when introduced at a given position in the S2 domain. Red indicates a particular mutation has a deleterious effect on binding and blue indicates that mutation has a neutral effect on binding. (*) show the proline substitutions, F817P and A942P introduced into the wild-type Wuhan-Hu-1 to stabilise protein expressions (37, 38). Boxed regions highlight elements of S2; fusion peptide (green), stem helix (purple) and heptad repeat 2 (yellow). G225 and G142 recognise the same epitope by competition SPR (**Figure 2C**) but are clonally unrelated (grey box) and their epitope is distributed over 3 distinct regions **(a–c)** separated in the linear sequence.

**Figure 5:**
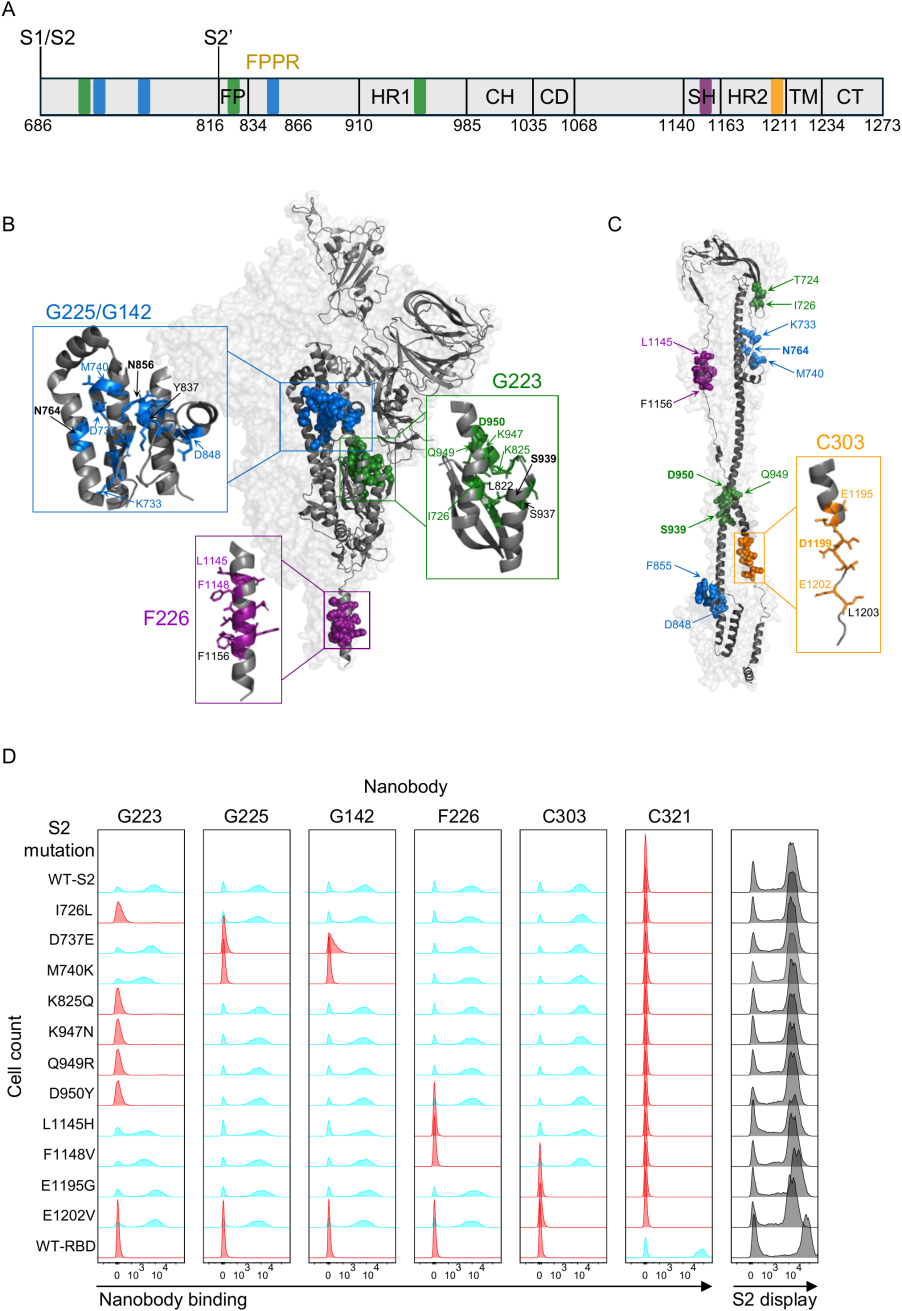
Correlating deep mutational scans with SARS-CoV-2 structure. **(A)** Schematic representation of the SARS-CoV-2 spike protein S2 elements showing the Nb epitopes on the linear sequence. Nb G223 as green bars, G225 & G142 as blue, F226 as purple and C303 as yellow bars. FP, fusion peptide; FPPR, fusion peptide proximal region; HR1, heptad repeat 1; CH, central helix region; CD, connector domain; SH, stem helix; HR2, heptad repeat 2; TM, transmembrane anchor; CT cytoplasmic tail. **(B)** Prefusion Wuhan-Hu-1 full trimeric spike (PDB: 7KRR) with one monomer highlighted in dark grey. Epitopes for Nb G223 (green), G225 & G142 (blue) and F226 (purple spheres) are highlighted on one monomer (49) **(C)** As the HR2 is not resolved in any available prefusion trimeric spike structures, the epitope for C303 is shown (yellow spheres) on one monomer of the postfusion S2 structure (PDB: 8FDW). This postfusion structure also shows the structural separation of the epitopes for G223, G225 and G142 in the postfusion conformation of S2. Amino acids D820-V826, region b of the G223 epitope, are not resolved in this crystal structure so not shown. In both **(B, C)**, mutations at the residue positions indicated in colour have been experimentally confirmed as reducing Nb binding. Residues in bold are naturally occurring resistance mutations that have also been tested experimentally for binding. **(D)** Flow cytometry histograms showing display of S2 clones containing single mutations identified by deep mutational scanning that escape binding of G223, G225, G142, F226 & C303 with the RBD binding Nb C321 as negative control. Cyan indicates the mutation has no impact on Nb binding, red indicates the mutation eliminates binding. Grey plots show S2 display.

The yeast display S2 mutant library was used to identify Nb epitopes through a combination of selection for escape from Nb binding followed by selection for correctly folded mutant S2 domains by selecting on a non-competing Nb (**Figure 3**) (29, 34). Comparison of the library to wild type S2 in flow cytometry showed that a high proportion of s2 sequences were still displayed on the yeast cell surface and .so folded sufficiently to pass through the yeast secretion apparatus (**Figure 3H**). For the first round of selection, 10 million cells, covering at least twice the library diversity, were labelled with each of the anti-S2 Nbs C303, F226, G223, G225 or G142 and the population of yeast cells containing S2 mutations that escape binding were collected (‘Escape fraction’ box **Figure 3D**). Escape populations were then sorted a second time using a non-competing Nb to identify only those mutations that specifically interfere with nanobody binding and eliminate pleiotropic mutations with non-specific effect on binding from the analysis (‘Folding reporter’ box **Figure 3E**). For example, C303 (epitope bin 1) was selected for escape from binding and then in the second round, on the non-competing nanobody F226 (epitope bin 2), from which the binding fraction was collected. All output libraries were analysed and single and double mutant frequencies relative to the unselected library were calculated to produce mutational scans and heatmaps of the escape fractions (**Figure 4**). The escape mutations were mapped onto a linear schematic of the SARS-CoV-2 spike protein, the prefusion full trimeric spike and postfusion S2 domain structures (**Figures 5A–C**) which showed the epitopes were non-overlapping. The G series epitopes were separated in the linear amino acid sequence (**Figure 5A**), but clustered together when mapped onto the spike protein structure (**Figure 5B**) making them conformational epitopes and indicating that S2 was displayed on the yeast cell surface in the prefusion conformation. The epitope of G223 maps to the fusion peptide, which is functionally important for membrane fusion during viral entry (1, 2) with overlap into heptad repeat 1. This region is the epitope for several human mAbs isolated previously from convalescent COVID-19 donors (16, 47). The epitopes of G142 and G225 had overlapping mutational scans, consistent with their being in the same epitope bin (**Figure 2C**), and bound regions either side of the fusion peptide. F226 bound to the highly conserved motif L1144-F1156 located in the stem helix region, and mAbs binding to this region have previously been isolated from convalescent COVID-19 donors (15, 17, 45). The stem helix goes through a major structural re-arrangement upon membrane fusion and is highly conserved amongst Sarbecoviruses (**Supplementary Figure S3**) (48). C303 maps to the heptad repeat 2 region close to the viral membrane but as this region is either disordered or not present in any available prefusions trimeric spike structures in PDB, we show the epitope of C303 mapped to the spike protein in the postfusion conformation (**Figure 5C**) (49). This region is functionally important and is also well conserved in all clades of Sarbecoviruses (**Supplementary Figure S3**) which is consistent with both F226 and C303 showing broad reactivity in ELISA (**Figure 2D**, **Supplementary Figure S2B**) and SPR (**Supplementary Figures S4A-I**). The epitopes of G225/G142 and G223 are well conserved in Clade 1b of the sarbecoviruses but less so for CoV-1 and the other betacoronaviruses with only G225 showing some binding to CoV-1 and binding to MERS was not detected by either of these Nbs (**Supplementary Figure S4**) as expected, given the sequence divergence from SARS-CoV-2 in these regions (**Supplementary Figure S3**). To confirm the yeast display deep mutational scans had identified relevant mutations, we used site-directed mutagenesis to validate single mutants by creating them in wild-type Wuhan-Hu-1 S2 background. We excluded any proline or cysteine mutations from analysis as they are likely to be structural with pleiotropic effects on Nb binding. Single mutations identified by DMS were tested for normalised binding to each of the five S2 Nbs in FACS (**Figure 5D**) and confirmed the elimination of binding only to the Nb predicted by the DMS.

### 3.5 Comparing mutational tolerance of two nanobodies binding to the same epitope

**Figure 6:**
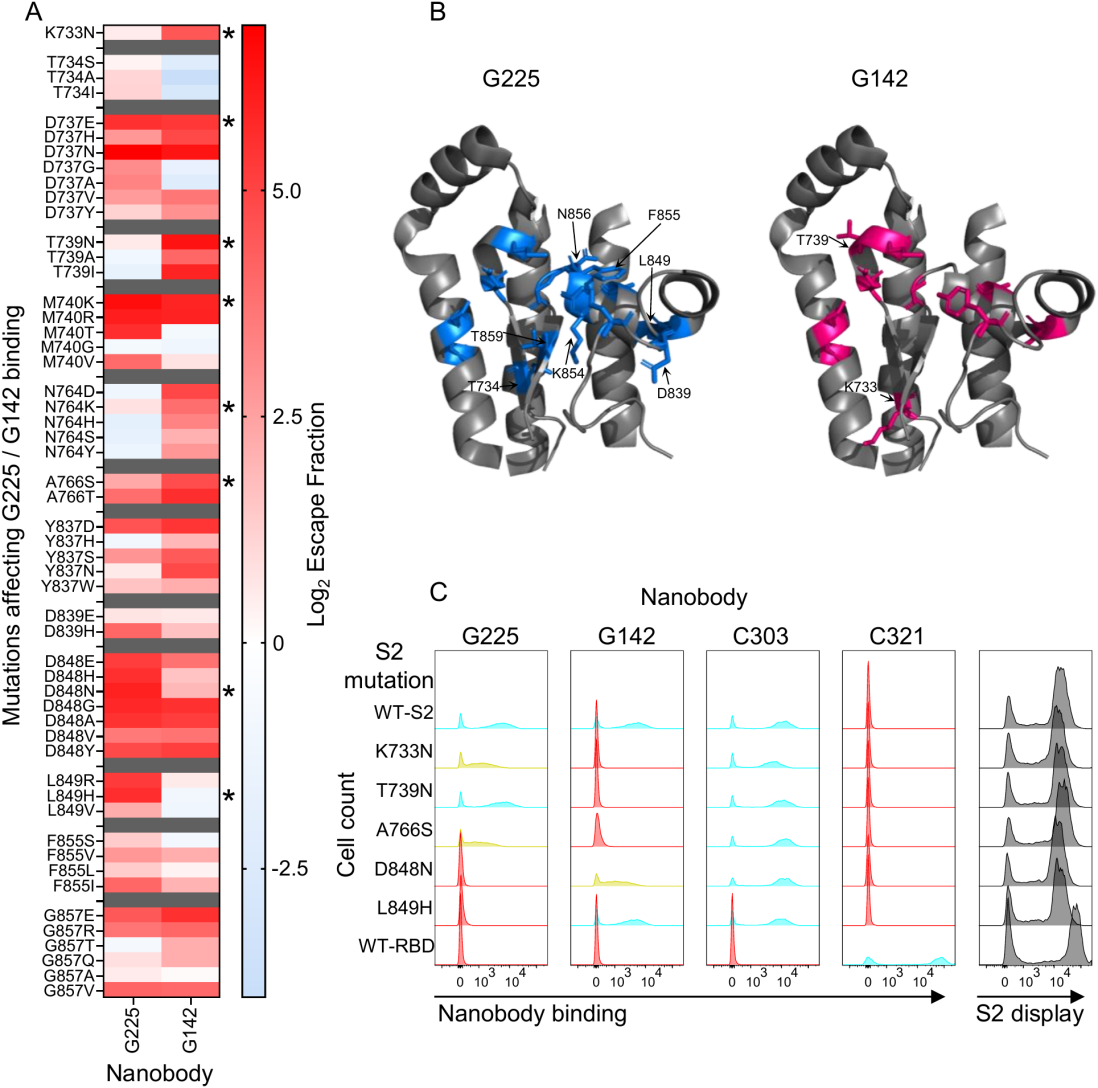
Comparing escape profiles of two nanobodies binding to the same epitope. **(A)** Alignment of mutational escape profiles and extraction of differences between G225 and G142 which bind to the same epitope. Red indicates mutation has a deleterious effect on binding and blue indicates no effect on binding. Mutations marked with * have been shown to lose binding in flow cytometry; D737E and M740K are shown in **Figure 5D**, N764K in **Figure 7C** and the rest in **Figure 6C**. **(B)** Magnified view of the escape mutations for G225 and G142 mapped onto the SARS-CoV-2 spike structure (PDB: 7KRR). **(C)** Flow cytometry histograms showing binding of a selection of the escape mutations. Cyan indicates the mutation has no impact on Nb binding, red indicates the mutation eliminates binding and yellow indicates an intermediate impact on Nb binding. Grey plots show S2 display.

**Figure 7:**
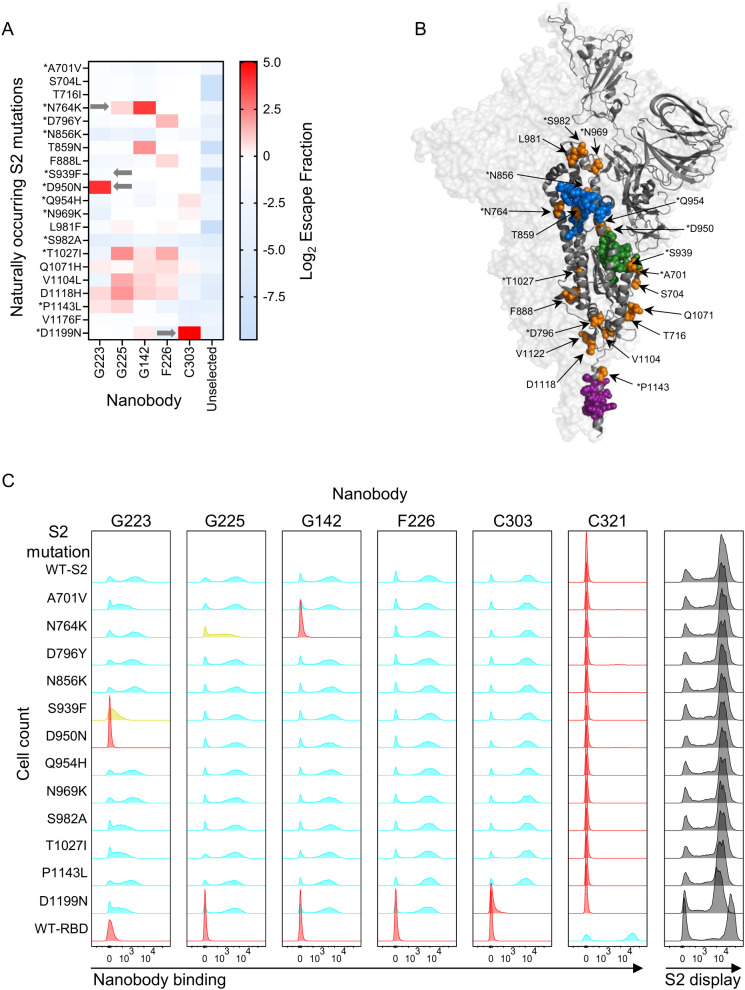
Application of deep mutational scanning to surveillance of antibody binding. **(A)** Extraction of escape fractions corresponding to naturally occurring S2 mutations obtained from deep mutational scanning of G223, G225, G142, F226 and C303. **(B)** Correlating naturally occurring S2 mutants with the epitopes of G223, G225/G142 and F226 on one monomer of prefusion spike. Amino acids marked with * are close to, or located within, a Nb epitope and were recreated for experimental testing **(C)** Naturally occurring S2 resistance mutations were produced as single amino acid mutants and displayed on yeast. Cyan indicates the mutation has no impact on Nb binding, red indicates the mutation eliminates binding and yellow indicates an intermediate impact on Nb binding. Grey plots show S2 display.

Although G225 and G142 belonged to the same epitope bin 3 (**Figure 2C**), comparing their hotspot maps revealed distinct differences in their mutational resistance (**Figure 6A**). This is not surprising as the nanobodies had clonally distinct CDR3 sequences and so different side chain interactions with the S2 domain. Most of the differences mapped in and around a common epitope footprint (**Figure 6B**). Several of the mutations (K733N, T739N, N764K, A766S, D848N & L849H) predicted to have a differential effect on binding of G225 and G142 were recreated by site-directed mutagenesis and tested in flow cytometry. Mutation T739N completely eliminated binding to G142 but had no effect on G225 as predicted by the deep mutational scanning. Conversely, L849H completely eliminated binding to G225 but did not affect G142 binding as predicted (**Figure 6C**). The other mutations recreated from **Figure 6A**, reduced or eliminated binding consistent with deep mutational scans (**Figure 5D** for D737E & M740K or **Figure 7C** for N764K).

### 3.6 Binding to S2 resistance mutations predicted by deep mutational scanning

To evaluate to what extent yeast display DMS can be used to predict antibody binding to the S2 domain, we extracted the escape fraction data for naturally occurring resistance mutations identified up to June 2024 in GISAID (50) (**Figure 7A**). We highlighted these mutations on one monomer of prefusion or postfusion spike along with each Nb epitope to show their proximity to each Nb epitope (**Figures 7B**, **5C**). Mutations close to, or located within, a Nb epitope were recreated in a Wuhan-Hu-1 S2 background and binding to Nb tested in flow cytometry. Yeast display DMS predicted that N764K, a mutation present in all Omicron variants, would affect G142 and G225 binding which was confirmed experimentally (**Figure 7C**). Further testing using SPR showed a >100x reduction in binding affinity for G225 and complete loss of binding of G142 to variants containing the N764K mutation (**Supplementary Figure S4**, **Table 1**). Mutation D950N, which emerged in the Delta (B.1.617) variant but then reverted back to the wild-type aspartate in Omicron lineages, was predicted to affect the binding of G223 and regain binding to post Delta sub-variants (**Figure 7A**). This was confirmed with binding of G223 to S2 domain carrying this mutation being eliminated in flow cytometry (**Figure 7C**), SPR (**Supplementary Figures S4B–E**) and ELISA (**Supplementary Figure S4J**) but was unaffected by Omicron variants where that mutation had reverted back to wild-type. S939F, a mutation present in the Omicron BA.2.86 variant and also located within the epitope of G223, was shown to specifically affect binding in flow cytometry (**Figure 7C**) and confirmed in SPR and ELISA on BA.2.86/Denmark which contains mutation S939F in combination with N764K (**Supplementary Figure S4E**). As the N764K mutation alone had no effect on binding of G223 (**Supplementary Figures S4C, J**), it was inferred that the S939F resistance mutation was leading to the loss of binding of G223 and not N764K in this variant. C303, which binds to the HR2 region, was predicted by DMS to be affected by the D1199N mutation found in the Omicron BA.2.75.2 variant. This transient loss of C303 binding was confirmed in flow cytometry (**Figure 7C**), ELISA and SPR to BA.2.75.2 full spike but no other Omicron variants that did not carry this mutation (**Supplementary Figures S4D, J**). Other natural resistance mutations that are located outside of the defined epitopes in all cases did not show any effect on Nb binding (**Figure 7C**). These retrospective examples clearly demonstrate that yeast displayed S2 domain libraries can provide useful surveillance data to predict which mutations could impact antibody binding should they emerge in future variants. Our data also reinforces that the S2 domain, whilst less susceptible to mutational escape than the RBD, is still prone to resistance mutations and presents a challenge to the development of durable anti-virals (21, 22).

### 3.7 Assessing combinations of mutations

We have used random error-prone PCR to construct our S2 library which contains both single and double mutant populations which can be independently analysed (**Supplementary Figure S1B**). Being able to analyse a library of unbiased combinations of mutations is valuable as synergistic mutations have been suggested to be an important driver of SARS-CoV-2 evolution (41, 42). To explore if any combinations of mutations had been selected, the double mutant component of the library were extracted and their frequency in the escape fraction compared to their frequency before selection. The enrichment of double mutants was then compared to the enrichment of each mutant individually within the single mutant population. The most frequent double mutants were mapped onto the SARS-CoV-2 structure to correlate with the respective nanobody epitopes (**Supplementary Figure S5**). This analysis identified combinations of mutations where both individual mutations co-locate within the epitope footprint of G142/G225 and G223, but no combinations of mutations could be identified for C303 or F226. Combinations of mutations with both synergistic effects and those with neutral effects were identified where only one of the mutations had a direct impact on Nb binding with the other being a ‘passenger’ mutation as identified by DMS. An example of synergistic combinations was A766T/T739N and A766T/T739I where both individual mutations lie in the epitope footprint for G225/G142 and both mutations individually and in combination led to loss of binding of G142. However, this combination of mutations had limited effect on G225 and represents a difference in the mutational resistance of these two nanobodies (**Figure 6**, **Supplementary Figure S5**). The D950N mutation, which emerged in the Delta (B.1.617) variant, was transient and reverted to wild type which correlated with restoration of G223 binding to more recent circulating variants. Of note was the enrichment of D950N in combination with over 5 other mutations including the mutation K825E/M/N. Both D950 and K825 lie in the epitope of G223 and are both associated with loss of binding as single mutants. D950N was also enriched with two other neutral mutations K921Q and S929R. Our study demonstrates error prone PCR is a useful addition to yeast display DMS and can lead to the identification of candidate functionally relevant combinations of mutations.

## 4 Discussion

Although yeast display DMS has been applied to the whole of the influenza HA molecule (29, 34), to date the technology has only been used to analyse mutations in the RBD domain of SARS-CoV-2 (31, 32). Deep mutational scanning using live virus on the whole spike protein or S2 domain has been limited to a pre-determined design of specific mutations (21, 28, 51). We have evaluated the potential of yeast display for unbiased deep mutational scanning of the whole of the conserved S2 domain. The S2 domain holds promise as a target for broad reactive anti-viral monoclonal antibodies and subunit vaccines however it is less well characterized in terms of the risks of resistance mutations. We describe an unbiased high-throughput evaluation of millions of single and double S2 mutations on binding of nanobodies to 4 different epitopes including important elements of the S2 domain, the stem helix (SH), fusion peptide (FP) and heptad repeat 2 (HR2) (36). We have used random mutagenesis to scan mutational effects on nanobody binding and have identified both single and candidate synergistic combinations of mutations which impacted G142/G225 and G223 binding. The identification of potential combinations of mutations was somewhat surprising given that we expected only a small fraction of the maximum theoretical combinatorial diversity of the S2 domain is captured within our library. Although yeast display DMS cannot directly correlate mutations with viral fitness or antibody neutralization and only uses antibody binding, it has been used successfully to map the epitopes of nanobodies against the conserved fusion peptide on the influenza stem which correlate with breadth of neutralization potency and *in vivo* efficacy (29, 34, 52, 53). The binding of antibodies to this highly conserved region of influenza have been difficult to map using conventional escape with live virus due to the virus being unable to fix mutations in this area without affecting viability (54) (10) which is not a limitation of yeast display DMS.

Prophylaxis or therapy with monoclonal antibodies against distinct S2 elements on SARS-CoV-2 spike is a promising addition to vaccines particularly in immunocompromised patients. However, it is not clear to what extent resistance mutations in the S2 domain may be a challenge to the development of such mAbs. We have correlated the maps generated against Nbs against important S2 elements like the FP, SH and HR2 domains by yeast display DMS with naturally occurring resistance mutations and experimentally tested if they correlate with their specificity profile. F226 binds to the stem helix (SH) and is the target of several other broad reactive antibodies (15, 45, 55, 56). Although this element is highly conserved across sarbecoviruses the identification of potential escape mutations indicates that the development of stem helix specific anti-viral or vaccines may still be susceptible to immune escape. C303 binds very close to the viral membrane in the HR2 domain which is also being explored as a target for broad reactive therapeutics and vaccines (35). To date we are not aware of any antibodies targeting this region, nevertheless we have shown it is still permissive to mutation which could eliminate C303 binding including the naturally occurring mutation D1199N which emerged transiently and then reverted back to wild-type (57). This mutation has been suggested to affect electrostatic repulsion resulting in a less upright spike protein which may impact binding to the ACE2 receptor (57). We are not aware of any antibodies where binding is lost and then regained in new sub-variants and speculate that this may be due to unique features of nanobody epitope compared to human antibodies. The fusion peptide (FP) is of considerable interest, and we have identified three nanobodies G142, G225 and G223 which bind in or around this element. Several human antibodies binding to this element have already been described with *in vivo* efficacy (16, 47, 58). The fusion peptide core has been shown previously to be susceptible to mutational escape and reduced viral fitness (21). Our yeast display DMS has confirmed the low mutational tolerance of this region and identified several naturally circulating resistance mutations in this region, N764K, S939F and D950N, the latter emerged in the Delta (B.1.617) variant but has reverted back to wild-type in more recent Omicron lineages. All these mutations were validated as eliminating or reducing binding of nanobodies G223, G225 and G142 to naturally occurring variants. The identification of S2 mutations that weaken binding of antibodies against the FP, SH and HR2 elements has implications for SARS-CoV-2 evolution and the development of universal vaccines and cautions that escape mutations may still pose a challenge for S2 based vaccines and anti-virals.

Out of the five nanobodies described in this study only F226, which binds in the stem helix, has not been impacted by naturally occurring S2 resistance mutations to date. Most of the existing antibodies which target this region are conventional human antibodies and it would be interesting to compare the mutational tolerance of these human antibodies against a nanobody to the same epitope. Nanobodies have considerable advantages over conventional antibodies including their ability to bind to clefts in proteins and easy reformatting into multi-paratopic molecules (52, 59). As all antigen binding is concentrated on a single domain they can be easily formatted for optimal mutational escape profiles, multi-specificity, effector functions, delivery and serum persistence (60). Having improved mAb based prophylactic measures with broad reactivity against betacoronavirus’s which are more tolerant of mutations will be useful for patient groups which do not respond well to vaccines.

Antibodies which target the RBD domain are highly potent but have proven to be prone to resistance mutations which emerged during the pandemic in new variants (7, 61–63). Antibodies which target the S2 domain have a greater breadth of reactivity and are more tolerant of mutational change but are considerably less potent in neutralizing virus which means the dose of S2 antibodies predicted to be required *in vivo* to be effective remains a challenge (15–17, 45). This trade off in potency against breadth of neutralization in antibodies which target the stem fusion peptide is also evident with influenza HA (10) and HIV (64). Nevertheless, the use of S2 nanobodies to different epitopes delivered either systemically, via nebulization directly into the lungs, or adenoviral mediated gene therapy warrants investigation as has been done for influenza (52, 65) and HIV (66). Durable expression from AAV gene therapy vectors coupled with new half-life extension technologies facilitated by the nanobody format may be able to achieve very high titres in serum to overcome some of these dose limitations. Animal model studies have shown that conventional S2 antibodies have a greater effect *in vivo* than is predicted by their *in vitro* neutralization titres so they may be more effective in patients than might be expected when compared to RBD antibodies (17, 45). Recent reports have highlighted the importance of non-neutralizing antibodies against the S2 domain which maintain binding to variant spikes and confer protection in animal models. Understanding the role of effector functions like antibody-dependent cellular cytotoxicity (ADCC) and antibody-dependent cellular phagocytosis (ADCP) in contributing to the overall effectiveness of S2 antibodies will be required to ensure their safe development (46).

When targeting variable pathogens, implementing yeast display DMS early in the antibody drug discovery process will aid in the choice of the most durable antibodies to progress to development. We have shown that antibodies binding to the same epitope (**Figure 6**) can have very different escape profiles. Additional early consideration should be given to selecting those antibodies most tolerant of mutation and as such present the greatest immune barrier for the virus to escape. By identifying S2 mutations that weaken binding to S2 specific antibodies and by identifying pathways of potential SARS-CoV-2 evolution it will be possible to mitigate this risk by engineering existing anti-viral monoclonal antibodies and subunit vaccines with compensatory changes against those high-risk resistance mutations. Yeast display DMS can produce huge datasets on mutational effects on antibody binding which could be used with machine learning and artificial intelligence to design compensatory changes in antibodies at pandemic pace.

## Data Availability

The datasets presented in this study can be found in online repositories. The name of repositories and accession numbers can be found here: PRJNA1377309 (SRA). All nanobody sequences are available on request.

## References

[1] V’Kovski P Kratzel A Steiner S Stalder H Thiel V . Coronavirus biology and replication: implications for SARS-CoV-2. Nat Rev Microbiol. (2021) 19:155–70. doi: 10.1038/s41579-020-00468-6, PMID: 33116300 PMC7592455

[2] Jackson CB Farzan M Chen B Choe H . Mechanisms of SARS-CoV-2 entry into cells. Nat Rev Mol Cell Biol. (2022) 23:3–20. doi: 10.1038/s41580-021-00418-x, PMID: 34611326 PMC8491763

[3] Wang Q Guo Y Iketani S Nair MS Li Z Mohri H . Antibody evasion by SARS-CoV-2 Omicron subvariants BA.2.12.1, BA.4 and BA.5. Nature. (2022) 608:603–8. doi: 10.1038/s41586-022-05053-w, PMID: 35790190 PMC9385487

[4] Cao Y Jian F Wang J Yu Y Song W Yisimayi A . Imprinted SARS-CoV-2 humoral immunity induces convergent Omicron RBD evolution. Nature. (2023) 614:521–9. doi: 10.1038/s41586-02205644-7, PMID: 36535326 PMC9931576

[5] Liu L Iketani S Guo Y Chan JF Wang M Liu L . Striking antibody evasion manifested by the Omicron variant of SARS-CoV-2. Nature. (2022) 602:676–81. doi: 10.1038/s41586-021-04388-0, PMID: 35016198

[6] Wang Q Iketani S Li Z Liu L Guo Y Huang Y . Alarming antibody evasion properties of rising SARS-CoV-2 BQ and XBB subvariants. Cell. (2023) 186:279–86.e8. doi: 10.1016/j.cell.2022.12.018, PMID: 36580913 PMC9747694

[7] Iketani S Liu L Guo Y Liu L Chan JF Huang Y . Antibody evasion properties of SARS-CoV-2 Omicron sublineages. Nature. (2022) 604:553–6. doi: 10.1038/s41586-022-04594-4, PMID: 35240676 PMC9021018

[8] Cao Y Wang J Jian F Xiao T Song W Yisimayi A . Omicron escapes the majority of existing SARS-CoV-2 neutralizing antibodies. Nature. (2022) 602:657–63. doi: 10.1038/s41586-021-04385-3, PMID: 35016194 PMC8866119

[9] Ekiert DC Bhabha G Elsliger MA Friesen RH Jongeneelen M Throsby M . Antibody recognition of a highly conserved influenza virus epitope. Science. (2009) 324:246–51. doi: 10.1126/science.1171491, PMID: 19251591 PMC2758658

[10] Throsby M van den Brink E Jongeneelen M Poon LL Alard P Cornelissen L . Heterosubtypic neutralizing monoclonal antibodies cross-protective against H5N1 and H1N1 recovered from human IgM+ memory B cells. PLoS One. (2008) 3:e3942. doi: 10.1371/journal.pone.0003942, PMID: 19079604 PMC2596486

[11] Zhou T Lynch RM Chen L Acharya P Wu X Doria-Rose NA . Structural repertoire of HIV-1-neutralizing antibodies targeting the CD4 supersite in 14 donors. Cell. (2015) 161:1280–92. doi: 10.1016/j.cell.2015.05.007, PMID: 26004070 PMC4683157

[12] Zhou T Xu L Dey B Hessell AJ Van Ryk D Xiang SH . Structural definition of a conserved neutralization epitope on HIV-1 gp120. Nature. (2007) 445:732–7. doi: 10.1038/nature05580, PMID: 17301785 PMC2584968

[13] Benton DJ Gamblin SJ Rosenthal PB Skehel JJ . Structural transitions in influenza haemagglutinin at membrane fusion pH. Nature. (2020) 583:150–3. doi: 10.1038/s41586-020-2333-6, PMID: 32461688 PMC7116728

[14] Skehel JJ Wiley DC . Receptor binding and membrane fusion in virus entry: the influenza hemagglutinin. Annu Rev Biochem. (2000) 69:531–69. doi: 10.1146/annurev.biochem.69.1.531, PMID: 10966468

[15] Pinto D Sauer MM Czudnochowski N Low JS Tortorici MA Housley MP . Broad betacoronavirus neutralization by a stem helix-specific human antibody. Science. (2021) 373:1109–16. doi: 10.1126/science.abj3321, PMID: 34344823 PMC9268357

[16] Dacon C Tucker C Peng L Lee CD Lin TH Yuan M . Broadly neutralizing antibodies target the coronavirus fusion peptide. Science. (2022) 377:728–35. doi: 10.1126/science.abq3773, PMID: 35857439 PMC9348754

[17] Zhou P Song G Liu H Yuan M He WT Beutler N . Broadly neutralizing anti-S2 antibodies protect against all three human betacoronaviruses that cause deadly disease. Immunity. (2023) 56:669–86.e7. doi: 10.1016/j.immuni.2023.02.005, PMID: 36889306 PMC9933850

[18] Sauer MM Tortorici MA Park YJ Walls AC Homad L Acton OJ . Structural basis for broad coronavirus neutralization. Nat Struct Mol Biol. (2021) 28:478–86. doi: 10.1038/s41594-021-00596-4, PMID: 33981021

[19] Wang C van Haperen R Gutierrez-Alvarez J Li W Okba NMA Albulescu I . A conserved immunogenic and vulnerable site on the coronavirus spike protein delineated by cross-reactive monoclonal antibodies. Nat Commun. (2021) 12:1715. doi: 10.1038/s41467-021-21968-w, PMID: 33731724 PMC7969777

[20] Li D Sempowski GD Saunders KO Acharya P Haynes BF . SARS-CoV-2 neutralizing antibodies for COVID-19 prevention and treatment. Annu Rev Med. (2022) 73:1–16. doi: 10.1146/annurev-med-042420-113838, PMID: 34428080

[21] Lei R Qing E Odle A Yuan M Gunawardene CD Tan TJC . Functional and antigenic characterization of SARS-CoV-2 spike fusion peptide by deep mutational scanning. Nat Commun. (2024) 15:4056. doi: 10.1038/s41467-024-48104-8, PMID: 38744813 PMC11094058

[22] Tan TJC Verma AK Odle A Lei R Meyerholz DK Matreyek KA . Evidence of antigenic drift in the fusion machinery core of SARS-CoV-2 spike. Proc Natl Acad Sci U S A. (2024) 121:e2317222121. doi: 10.1073/pnas.2317222121, PMID: 38557175 PMC11009667

[23] Kumar R Srivastava Y Muthuramalingam P Singh SK Verma G Tiwari S . Understanding mutations in human SARS-CoV-2 spike glycoprotein: A systematic review & Meta-analysis. Viruses. (2023) 15:856. doi: 10.3390/v15040856, PMID: 37112836 PMC10142771

[24] Nakamura G Chai N Park S Chiang N Lin Z Chiu H . An *in vivo* human-plasmablast enrichment technique allows rapid identification of therapeutic influenza A antibodies. Cell Host Microbe. (2013) 14:93–103. doi: 10.1016/j.chom.2013.06.004, PMID: 23870317

[25] Anderson CS Ortega S Chaves FA Clark AM Yang H Topham DJ . Natural and directed antigenic drift of the H1 influenza virus hemagglutinin stalk domain. Sci Rep. (2017) 7:14614. doi: 10.1038/s41598-017-14931-7, PMID: 29097696 PMC5668287

[26] Chai N Swem LR Reichelt M Chen-Harris H Luis E Park S . Two escape mechanisms of influenza A virus to a broadly neutralizing stalk-binding antibody. PLoS Pathog. (2016) 12:e1005702. doi: 10.1371/journal.ppat.1005702, PMID: 27351973 PMC4924800

[27] Narayanan KK Procko E . Deep mutational scanning of viral glycoproteins and their host receptors. Front Mol Biosci. (2021) 8:636660. doi: 10.3389/fmolb.2021.636660, PMID: 33898517 PMC8062978

[28] Dadonaite B Crawford KHD Radford CE Farrell AG Yu TC Hannon WW . A pseudovirus system enables deep mutational scanning of the full SARS-CoV-2 spike. Cell. (2023) 186:1263–78.e20. doi: 10.1016/j.cell.2023.02.001, PMID: 36868218 PMC9922669

[29] Gaiotto T Hufton SE . Cross-neutralising nanobodies bind to a conserved pocket in the hemagglutinin stem region identified using yeast display and deep mutational scanning. PLoS One. (2016) 11:e0164296. doi: 10.1371/journal.pone.0164296, PMID: 27741319 PMC5065140

[30] Starr TN Greaney AJ Stewart CM Walls AC Hannon WW Veesler D . Deep mutational scans for ACE2 binding, RBD expression, and antibody escape in the SARS-CoV-2 Omicron BA.1 and BA.2 receptor-binding domains. PLoS Pathog. (2022) 18:e1010951. doi: 10.1371/journal.ppat.1010951, PMID: 36399443 PMC9674177

[31] Greaney AJ Starr TN Gilchuk P Zost SJ Binshtein E Loes AN . Complete mapping of mutations to the SARS-CoV-2 spike receptor-binding domain that escape antibody recognition. Cell Host Microbe. (2021) 29:44–57.e9. doi: 10.1016/j.chom.2020.11.007, PMID: 33259788 PMC7676316

[32] Starr TN Greaney AJ Hilton SK Ellis D Crawford KHD Dingens AS . Deep mutational scanning of SARS-CoV-2 receptor binding domain reveals constraints on folding and ACE2 binding. Cell. (2020) 182:1295–310.e20. doi: 10.1016/j.cell.2020.08.012, PMID: 32841599 PMC7418704

[33] Chan KK Tan TJC Narayanan KK Procko E . An engineered decoy receptor for SARS-CoV-2 broadly binds protein S sequence variants. Sci Adv. (2021) 7. doi: 10.1126/sciadv.abf1738, PMID: 33597251 PMC7888922

[34] Gaiotto T Ramage W Ball C Risley P Carnell G Temperton N . Nanobodies mapped to cross-reactive and divergent epitopes on A(H7N9) influenza hemagglutinin using yeast display. Sci Rep. (2021) 11:3126. doi: 10.1038/s41598-021-82356-4, PMID: 33542302 PMC7862619

[35] Xia S Zhu Y Liu M Lan Q Xu W Wu Y . Fusion mechanism of 2019-nCoV and fusion inhibitors targeting HR1 domain in spike protein. Cell Mol Immunol. (2020) 17:765–7. doi: 10.1038/s41423-020-0374-2, PMID: 32047258 PMC7075278

[36] Guo L Lin S Chen Z Cao Y He B Lu G . Targetable elements in SARS-CoV-2 S2 subunit for the design of pan-coronavirus fusion inhibitors and vaccines. Signal Transduct Target Ther. (2023) 8:197. doi: 10.1038/s41392-023-01472-x, PMID: 37164987 PMC10170451

[37] Hsieh CL Goldsmith JA Schaub JM DiVenere AM Kuo HC Javanmardi K . Structure-based design of prefusion-stabilized SARS-CoV-2 spikes. Science. (2020) 369:1501–5. doi: 10.1126/science.abd0826, PMID: 32703906 PMC7402631

[38] Wrapp D Wang N Corbett KS Goldsmith JA Hsieh CL Abiona O . Cryo-EM structure of the 2019-nCoV spike in the prefusion conformation. Science. (2020) 367:1260–3. doi: 10.1126/science.abb2507, PMID: 32075877 PMC7164637

[39] Chao G Lau WL Hackel BJ Sazinsky SL Lippow SM Wittrup KD . Isolating and engineering human antibodies using yeast surface display. Nat Protoc. (2006) 1:755–68. doi: 10.1038/nprot.2006.94, PMID: 17406305

[40] Hufton SE Risley P Ball CR Major D Engelhardt OG Poole S . The breadth of cross sub-type neutralisation activity of a single domain antibody to influenza hemagglutinin can be increased by antibody valency. PLoS One. (2014) 9:e103294. doi: 10.1371/journal.pone.0103294, PMID: 25084445 PMC4118869

[41] Cox M Peacock TP Harvey WT Hughes J Wright DW Consortium C-GU . SARS-CoV-2 variant evasion of monoclonal antibodies based on *in vitro* studies. Nat Rev Microbiol. (2023) 21:112–24. doi: 10.1038/s41579-022-00809-7, PMID: 36307535 PMC9616429

[42] Carabelli AM Peacock TP Thorne LG Harvey WT Hughes J Consortium C-GU . SARS-CoV-2 variant biology: immune escape, transmission and fitness. Nat Rev Microbiol. (2023) 21:162–77. doi: 10.1038/s41579-022-00841-7, PMID: 36653446 PMC9847462

[43] Ferrara F Temperton N . Pseudotype neutralization assays: from laboratory bench to data analysis. Methods Protoc. (2018) 1. doi: 10.3390/mps1010008, PMID: 31164554 PMC6526431

[44] Clark JJ Hoxie I Adelsberg DC Sapse IA Andreata-Santos R Yong JS . Protective effect and molecular mechanisms of human non-neutralizing cross-reactive spike antibodies elicited by SARS-CoV-2 mRNA vaccination. Cell Rep. (2024) 43:114922. doi: 10.1016/j.celrep.2024.114922, PMID: 39504245 PMC11804229

[45] Zhou P Yuan M Song G Beutler N Shaabani N Huang D . A human antibody reveals a conserved site on beta-coronavirus spike proteins and confers protection against SARS-CoV-2 infection. Sci Transl Med. (2022) 14:eabi9215. doi: 10.1126/scitranslmed.abi9215, PMID: 35133175 PMC8939767

[46] Schafer A Muecksch F Lorenzi JCC Leist SR Cipolla M Bournazos S . Antibody potency, effector function, and combinations in protection and therapy for SARS-CoV-2 infection *in vivo*. J Exp Med. (2021) 218. doi: 10.1084/jem.20201993, PMID: 33211088 PMC7673958

[47] Low JS Jerak J Tortorici MA McCallum M Pinto D Cassotta A . ACE2-binding exposes the SARS-CoV-2 fusion peptide to broadly neutralizing coronavirus antibodies. Science. (2022) 377:735–42. doi: 10.1126/science.abq2679, PMID: 35857703 PMC9348755

[48] Yang K Wang C White KI Pfuetzner RA Esquivies L Brunger AT . Structural conservation among variants of the SARS-CoV-2 spike postfusion bundle. Proc Natl Acad Sci U.S.A. (2022) 119:e2119467119. doi: 10.1073/pnas.2119467119, PMID: 35363556 PMC9169775

[49] Shi W Cai Y Zhu H Peng H Voyer J Rits-Volloch S . Cryo-EM structure of SARS-CoV-2 postfusion spike in membrane. Nature. (2023) 619:403–9. doi: 10.1038/s41586-023-06273-4, PMID: 37285872

[50] Khare S Gurry C Freitas L Schultz MB Bach G Diallo A . GISAID’s role in pandemic response. China CDC Wkly. (2021) 3:1049–51. doi: 10.46234/ccdcw2021.255, PMID: 34934514 PMC8668406

[51] Javanmardi K Chou CW Terrace CI Annapareddy A Kaoud TS Guo Q . Rapid characterization of spike variants via mammalian cell surface display. Mol Cell. (2021) 81:5099–111.e8. doi: 10.1016/j.molcel.2021.11.024, PMID: 34919820 PMC8675084

[52] Del Rosario JMM Smith M Zaki K Risley P Temperton N Engelhardt OG . Protection from influenza by intramuscular gene vector delivery of a broadly neutralizing nanobody does not depend on antibody dependent cellular cytotoxicity. Front Immunol. (2020) 11:627. doi: 10.3389/fimmu.2020.00627, PMID: 32547534 PMC7273724

[53] Ramage W Gaiotto T Ball C Risley P Carnell GW Temperton N . Cross-reactive and lineage-specific single domain antibodies against influenza B hemagglutinin. Antibodies (Basel). (2019) 8. doi: 10.3390/antib8010014, PMID: 31544820 PMC6640691

[54] Okuno Y Isegawa Y Sasao F Ueda S . A common neutralizing epitope conserved between the hemagglutinins of influenza A virus H1 and H2 strains. J Virol. (1993) 67:2552–8. doi: 10.1128/jvi.67.5.2552-2558.1993, PMID: 7682624 PMC237575

[55] Bianchini F Crivelli V Abernathy ME Guerra C Palus M Muri J . Human neutralizing antibodies to cold linear epitopes and subdomain 1 of the SARS-CoV-2 spike glycoprotein. Sci Immunol. (2023) 8:eade0958. doi: 10.1126/sciimmunol.ade0958, PMID: 36701425 PMC9972897

[56] Hurlburt NK Homad LJ Sinha I Jennewein MF MacCamy AJ Wan YH . Structural definition of a pan-sarbecovirus neutralizing epitope on the spike S2 subunit. Commun Biol. (2022) 5:342. doi: 10.1038/s42003-022-03262-7, PMID: 35411021 PMC9001700

[57] Qu P Evans JP Faraone JN Zheng YM Carlin C Anghelina M . Enhanced neutralization resistance of SARS-CoV-2 Omicron subvariants BQ.1, BQ.1.1, BA.4.6, BF.7, and BA.2.75.2. Cell Host Microbe. (2023) 31:9–17.e3. doi: 10.1016/j.chom.2022.11.012, PMID: 36476380 PMC9678813

[58] Sun X Yi C Zhu Y Ding L Xia S Chen X . Neutralization mechanism of a human antibody with pan-coronavirus reactivity including SARS-CoV-2. Nat Microbiol. (2022) 7:1063–74. doi: 10.1038/s41564-022-01155-3, PMID: 35773398

[59] Muyldermans S . Nanobodies: natural single-domain antibodies. Annu Rev Biochem. (2013) 82:775–97. doi: 10.1146/annurev-biochem-063011-092449, PMID: 23495938

[60] Muyldermans S Baral TN Retamozzo VC De Baetselier P De Genst E Kinne J . Camelid immunoglobulins and nanobody technology. Vet Immunol Immunopathol. (2009) 128:178–83. doi: 10.1016/j.vetimm.2008.10.299, PMID: 19026455

[61] VanBlargan LA Errico JM Halfmann PJ Zost SJ Crowe JE Jr. Purcell LA . An infectious SARS-CoV-2 B.1.1.529 Omicron virus escapes neutralization by therapeutic monoclonal antibodies. Nat Med. (2022) 28:490–5. doi: 10.1038/s41591-021-01678-y, PMID: 35046573 PMC8767531

[62] Takashita E Yamayoshi S Simon V van Bakel H Sordillo EM Pekosz A . Efficacy of antibodies and antiviral drugs against omicron BA.2.12.1, BA.4, and BA.5 subvariants. N Engl J Med. (2022) 387:468–70. doi: 10.1056/NEJMc2207519, PMID: 35857646 PMC9342381

[63] Tortorici MA Czudnochowski N Starr TN Marzi R Walls AC Zatta F . Broad sarbecovirus neutralization by a human monoclonal antibody. Nature. (2021) 597:103–8. doi: 10.1038/s41586-021-03817-4, PMID: 34280951 PMC9341430

[64] Kong R Xu K Zhou T Acharya P Lemmin T Liu K . Fusion peptide of HIV-1 as a site of vulnerability to neutralizing antibody. Science. (2016) 352:828–33. doi: 10.1126/science.aae0474, PMID: 27174988 PMC4917739

[65] Laursen NS Friesen RHE Zhu X Jongeneelen M Blokland S Vermond J . Universal protection against influenza infection by a multidomain antibody to influenza hemagglutinin. Science. (2018) 362:598–602. doi: 10.1126/science.aaq0620, PMID: 30385580 PMC6241527

[66] Balazs AB Chen J Hong CM Rao DS Yang L Baltimore D . Antibody-based protection against HIV infection by vectored immunoprophylaxis. Nature. (2011) 481:81–4. doi: 10.1038/nature10660, PMID: 22139420 PMC3253190

