# Supplementary material for: Susceptibility of broad reactivity nanobodies to resistance mutations in the S2 domain of SARS-CoV-2 predicted by yeast display deep mutational scanning
Source: Front Immunol. 2026 Jan 12;16:1726449. doi: 10.3389/fimmu.2025.1726449 (PMC12832929; doi:10.3389/fimmu.2025.1726449)
Supplement: Supplementary file 1 [file DataSheet1.pdf]

Supplementary Material

Supplementary Fig S1

A

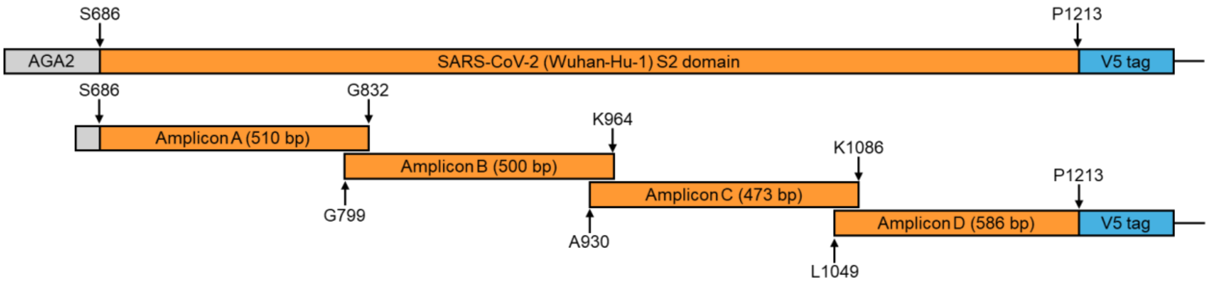

B

	S2 domain library diversity			
	**Amplicon A**	**Amplicon B**	**Amplicon C**	**Amplicon D**
Total number of correct length paired reads	249,343	302,906	376,778	148,367
Total number of wild-type sequences (% of total reads)	165,232 (66%)	180,013 (59%)	261,346 (69%)	58,950 (40%)
Total number of single mutants (% of total reads)	59,681 (24%)	82,276 (27%)	88,599 (23.5%)	45,083 (30%)
Total number of double mutants (% of total reads)	3,628 (1.4%)	26,721 (9%)	20,562 (5.4%)	23,601 (16%)
Total number of unique single amino acid mutants	1113	1252	1060	1020
Length of amplicon (without overlap)	147 aa	132 aa	122 aa	127 aa
Max theoretical number of amino acid residues	2940	2640	2440	2540
Percentage unique single mutant diversity per amplicon	38%	47%	43%	40%

Average diversity of unique single mutants per amplicon in S2 domain library = 43%

C

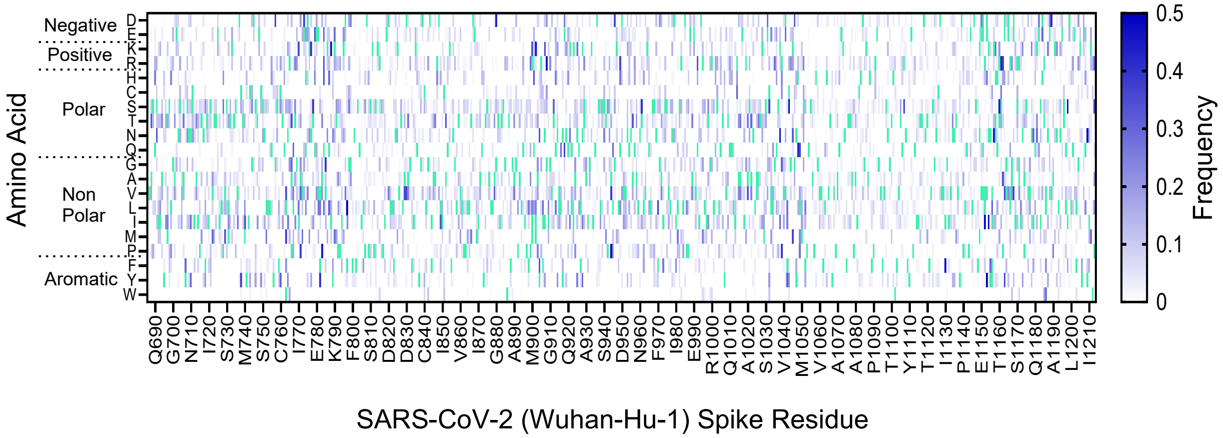

Supplementary Fig S1. Assessment of diversity of S2 domain library. (A) The S2 library split into 4 amplicons for NGS sequencing. **(B)** Analysis of sequencing reads containing wild-type, single and double mutants. **(C)** Deep mutational scanning results of the unselected single mutant population of the S2 library plotted as a heatmap with S2 sequence on the x-axis and individual amino acids on the y-axis. The wild type amino acids at each position are coloured in green and blue is shaded according to frequency in the library.

Supplementary Fig S2

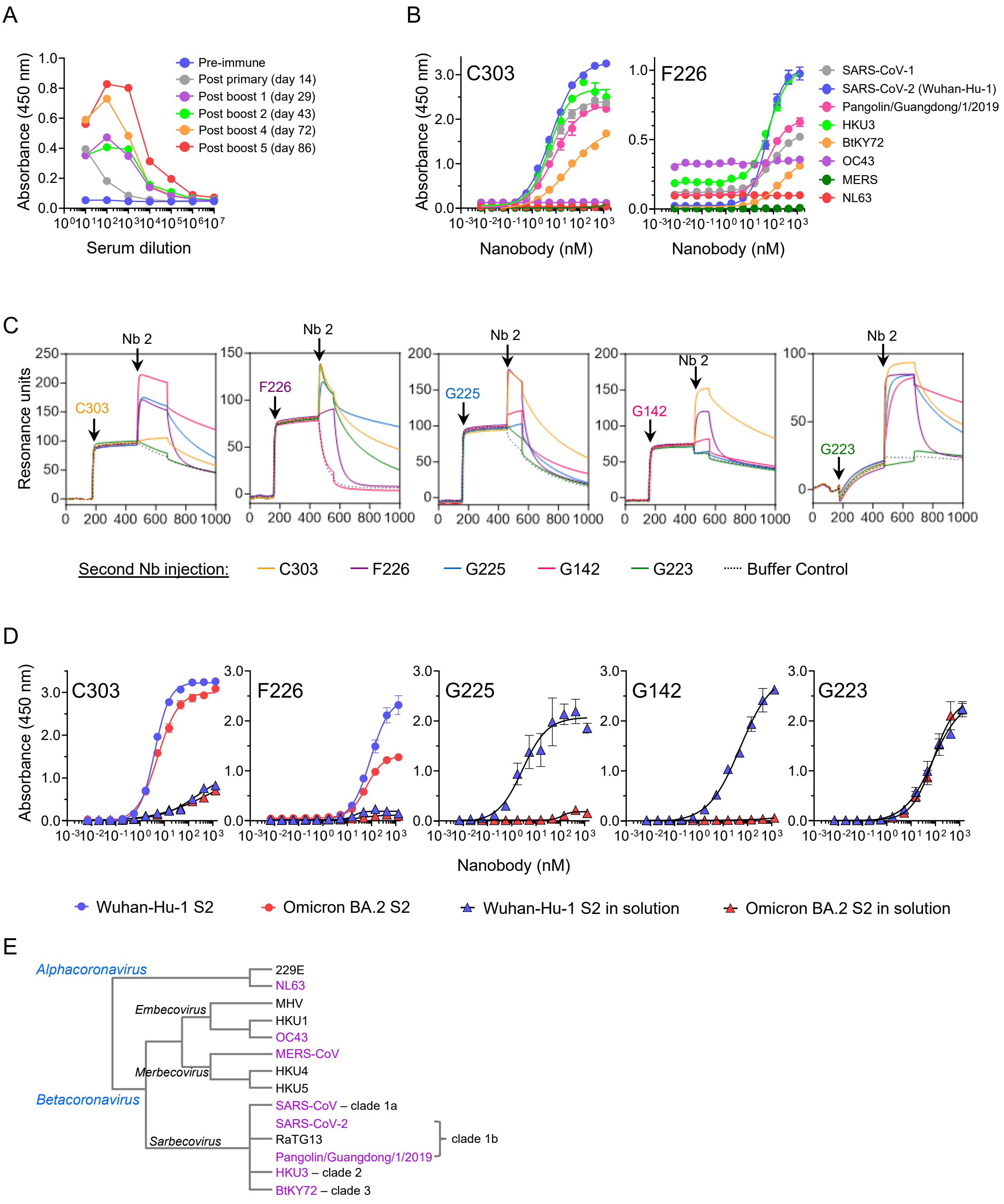

Supplementary Fig S2. Isolation and characterisation of broad reactivity nanobodies against the S2 domain of SARS-CoV-2. (A) Alpaca serological response to Wuhan-Hu-1 SARS-CoV-2 spike trimer. **(B)** Binding of Nbs C303 and F226 to full trimeric spike coated on an ELISA plate showing broad reactivity against all clades of sarbecovirus. **(C)** Five S2 specific Nbs with broad reactivity and unique CDR3 sequences were tested in combination to identify non-competing nanobody pairs using competition Surface Plasmon Resonance (SPR). **(D)** Binding of Nbs to SARS-CoV-2 strains Wuhan-Hu-1 or Omicron BA.2 S2 domain in solution and Nbs F226 and C303 to the same strains immobilised on the plate. **(E)** Diagram of phylogentic relatedness of alpha- and beta-coronavirus S protein. Trimeric spike proteins shown in purple have been tested for binding to Nbs F226 & C303.

Supplementary Fig S3

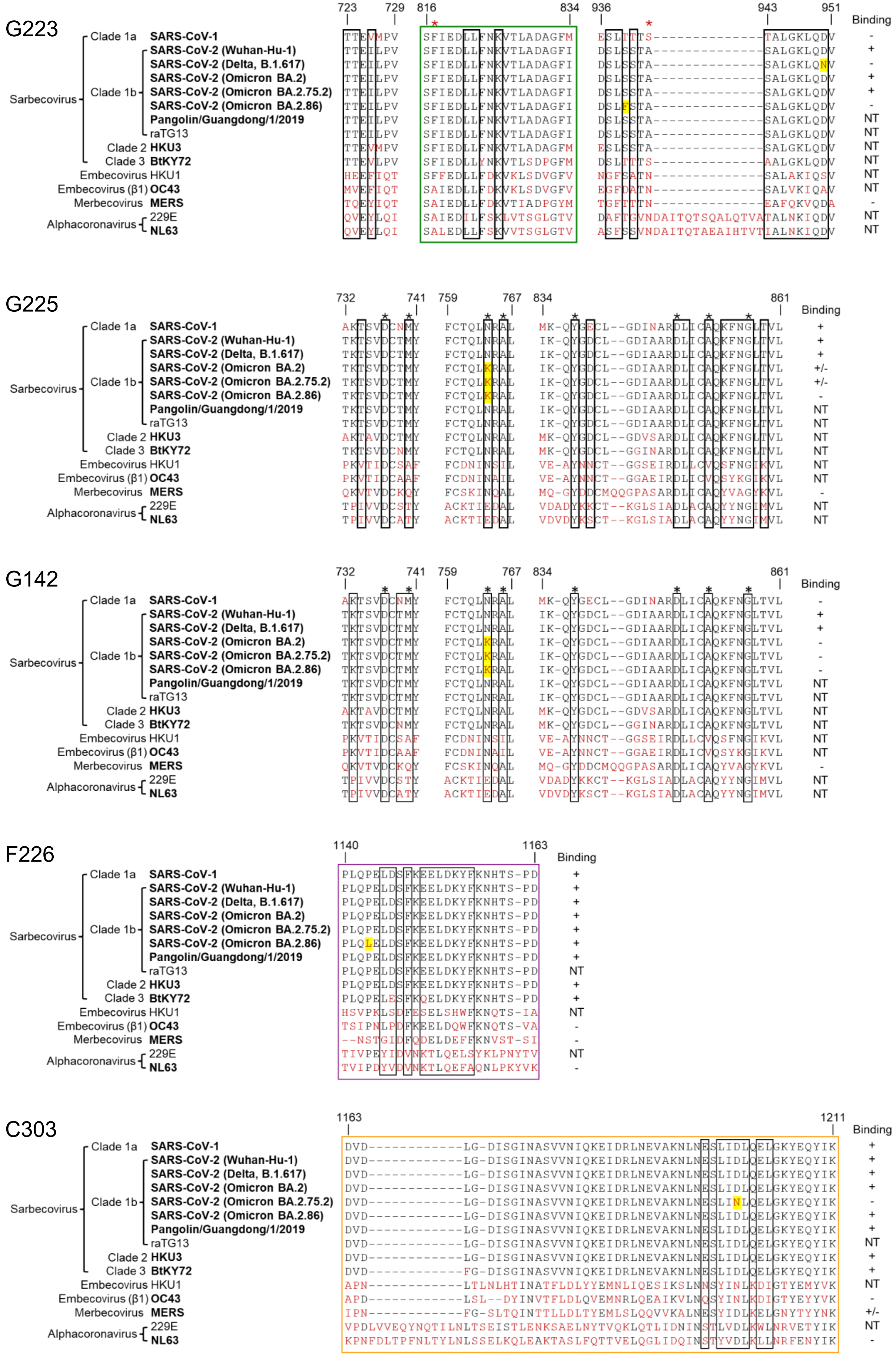

Supplementary Fig S3. Conservation of Alpha- and Beta-coronavirus amino acids correlated with Nb epitope. Numbering is for Wuhan-Hu-1 SARS-CoV-2 with Nb epitopes boxed in black. (*) in the G223 epitope alignment show the proline substitutions F817P and A942P we introduced into the wild-type Wuhan-Hu-1 to stabilise protein expressions [36,37] on the yeast cell surface. Residue positions common to the epitopes of both G225 & G142 are labelled (*). Identical amino acids with Wuhan-Hu-1 S2 are in black, alterations in red and natural SARS-CoV-2 mutations that have emerged since the pandemic are highlighted in yellow. Coloured boxed regions highlight functionally important regions of S2; fusion peptide, green; stem helix, purple; and heptad repeat 2, yellow. Spike proteins in bold were tested for binding in ELISA (Fig 2B and 2D & S2B) or SPR (Fig S4A-I) and the results summarised here. The '+/-' indicates that binding has decreased significantly with that variant. Sequences were aligned in Geneious Prime version 2020.0.3.

Supplementary Fig S4

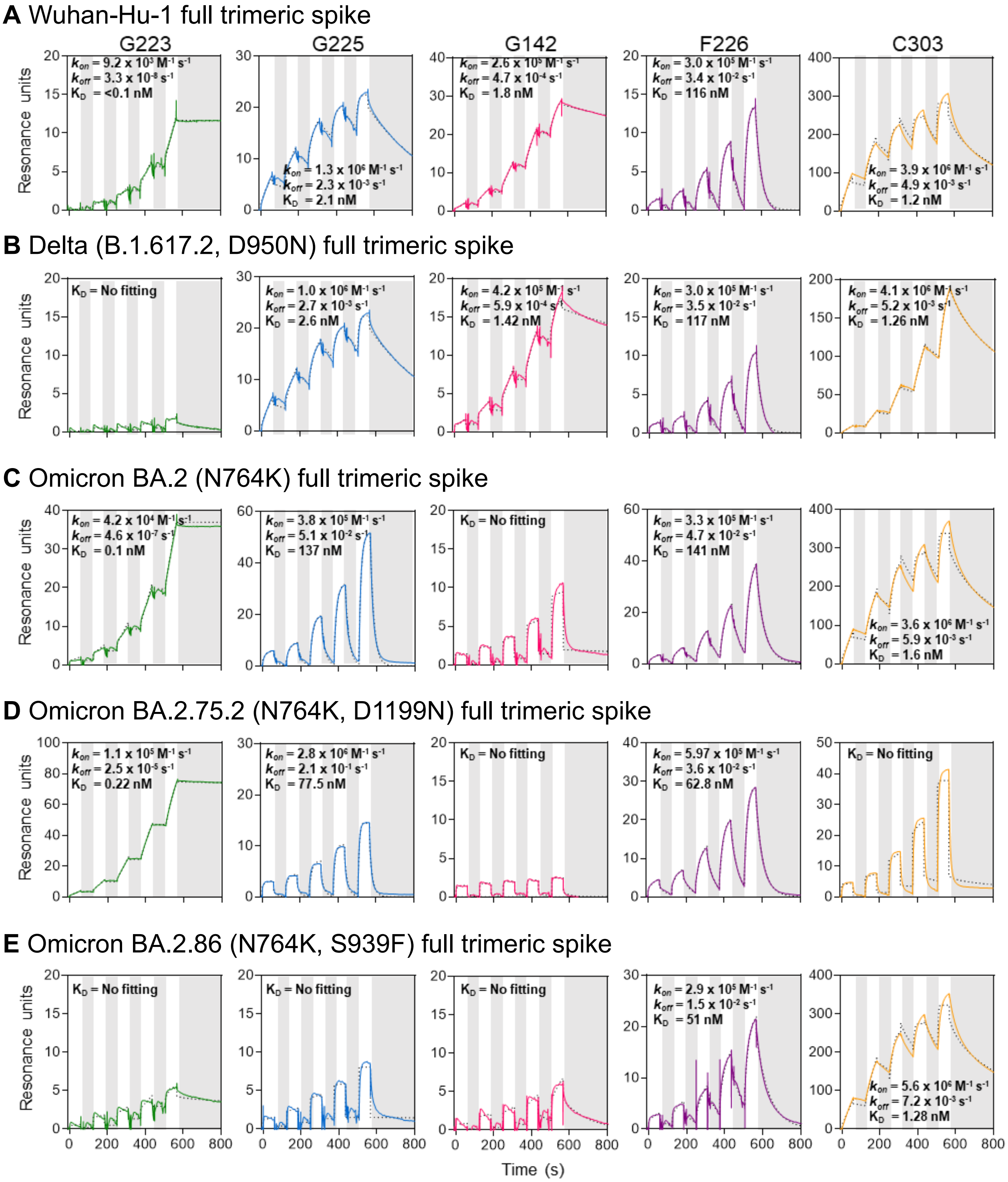

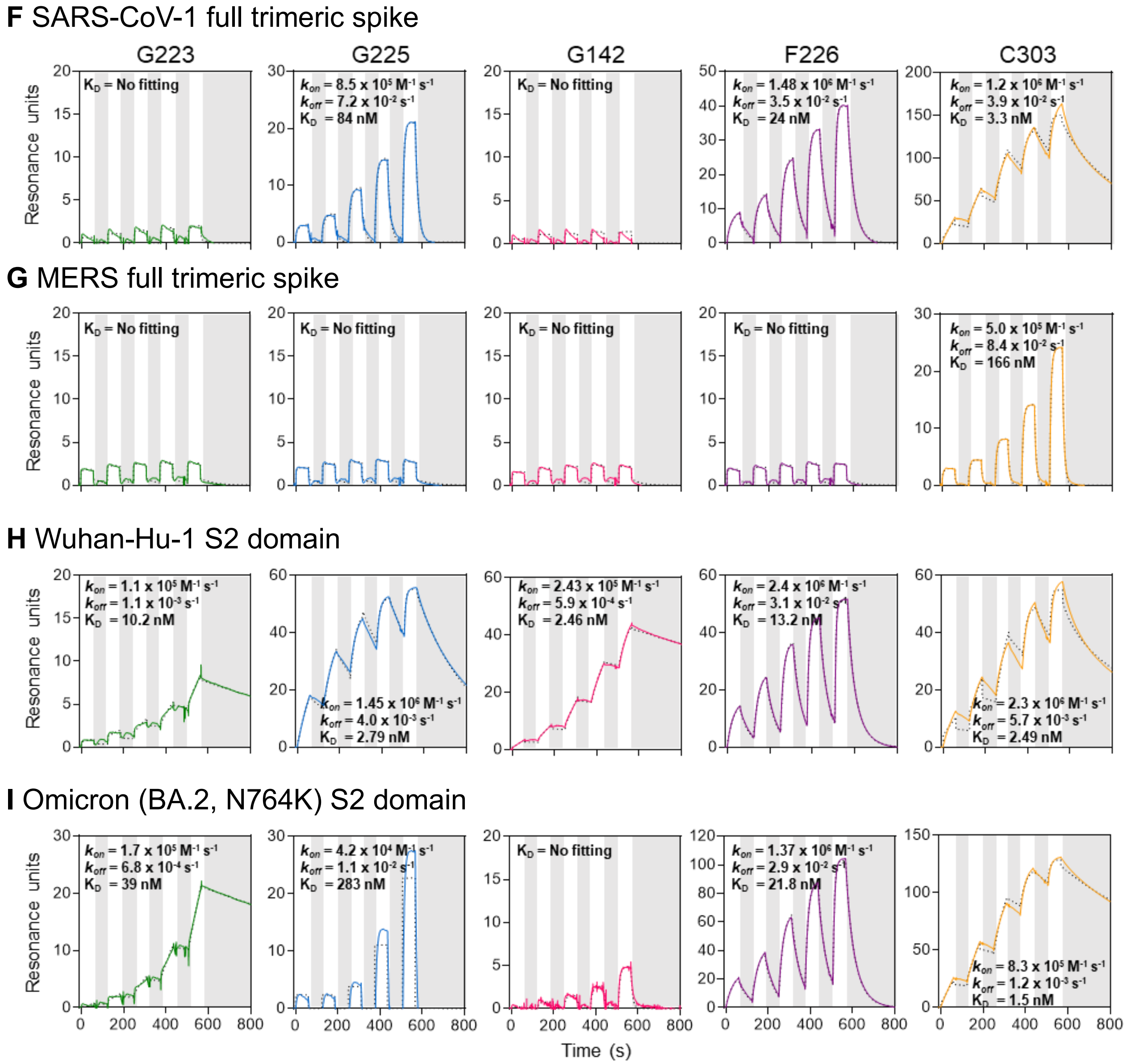
F SARS-CoV-1 full trimeric spike
G223
G225
G142
F226
C303
K_D = No fitting
k_on = 8.5 x 10^5 M^-1 s^-1
k_off = 7.2 x 10^-2 s^-1
K_D = 84 nM
K_D = No fitting
k_on = 1.48 x 10^6 M^-1 s^-1
k_off = 3.5 x 10^-2 s^-1
K_D = 24 nM
k_on = 1.2 x 10^6 M^-1 s^-1
k_off = 3.9 x 10^-3 s^-1
K_D = 3.3 nM
G MERS full trimeric spike
K_D = No fitting
K_D = No fitting
K_D = No fitting
K_D = No fitting
k_on = 5.0 x 10^5 M^-1 s^-1
k_off = 8.4 x 10^-2 s^-1
K_D = 166 nM
H Wuhan-Hu-1 S2 domain
k_on = 1.1 x 10^5 M^-1 s^-1
k_off = 1.1 x 10^-3 s^-1
K_D = 10.2 nM
k_on = 1.45 x 10^6 M^-1 s^-1
k_off = 4.0 x 10^-3 s^-1
K_D = 2.79 nM
k_on = 2.43 x 10^5 M^-1 s^-1
k_off = 5.9 x 10^-4 s^-1
K_D = 2.46 nM
k_on = 2.4 x 10^6 M^-1 s^-1
k_off = 3.1 x 10^-2 s^-1
K_D = 13.2 nM
k_on = 2.3 x 10^6 M^-1 s^-1
k_off = 5.7 x 10^-3 s^-1
K_D = 2.49 nM
I Omicron (BA.2, N764K) S2 domain
k_on = 1.7 x 10^5 M^-1 s^-1
k_off = 6.8 x 10^-4 s^-1
K_D = 39 nM
k_on = 4.2 x 10^4 M^-1 s^-1
k_off = 1.1 x 10^-2 s^-1
K_D = 283 nM
K_D = No fitting
k_on = 1.37 x 10^6 M^-1 s^-1
k_off = 2.9 x 10^-2 s^-1
K_D = 21.8 nM
k_on = 8.3 x 10^5 M^-1 s^-1
k_off = 1.2 x 10^-3 s^-1
K_D = 1.5 nM
Resonance units
Time (s)

J

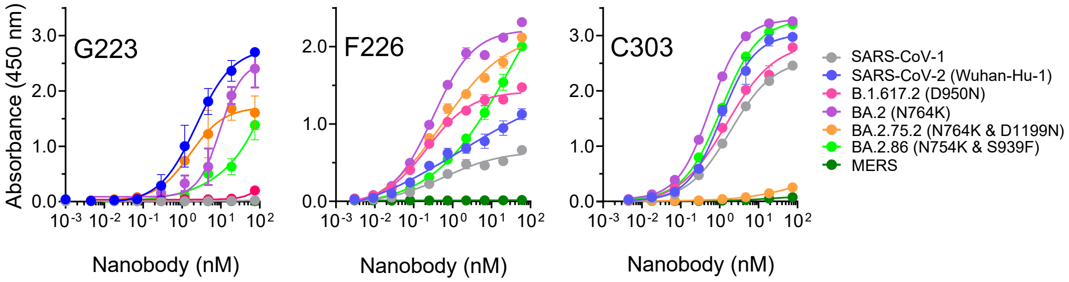

	EC_{50} (nM)		
**Coronavirus strain**	**G223**	**F226**	**C303**
SARS-CoV-1	-	0.58	1.91
SARS-CoV-2 (Wuhan-Hu-1)	2.31	1.55	1.1
SARS-CoV-2 (B.1.617.2, D950N)	-	0.23	1.52
SARS-CoV-2 (BA.2, N764K)	9.57	0.32	0.56
SARS-CoV-2 (BA.2.75.2, N764K & D1199N)	1.62	0.55	160.1
SARS-CoV-2 (BA.2.86, N764K & S939F)	$	30.64	1.07
MERS	-	-	$

Supplementary Fig S4. Surveillance of nanobody binding to SARS-CoV-1, natural mutations in full spike and S2 domain of SARS-CoV-2 and MERS. (A-I) Affinity determination using single cycle kinetics on biotinylated antigens immobilised on a streptavidin coated chip. White and grey stripes indicate association and dissociation phases respectively. Nb G223 binding to full trimeric spikes was evaluated using G223-IgG1 Fc fusion. **(J)** Binding curves of Nb-Fc fusions G223, F226 and C303 to full trimeric spikes of SARS-CoV-1, natural variants of SARS-CoV-2 and MERS immobilised via a C-terminal biotin tag with table showing EC_{50} values determined by GraphPad Prism 10 software, from the ELISA curves. '–' indicates no binding and '$' indicates the EC_{50} could not be determined.

Supplementary Fig S5

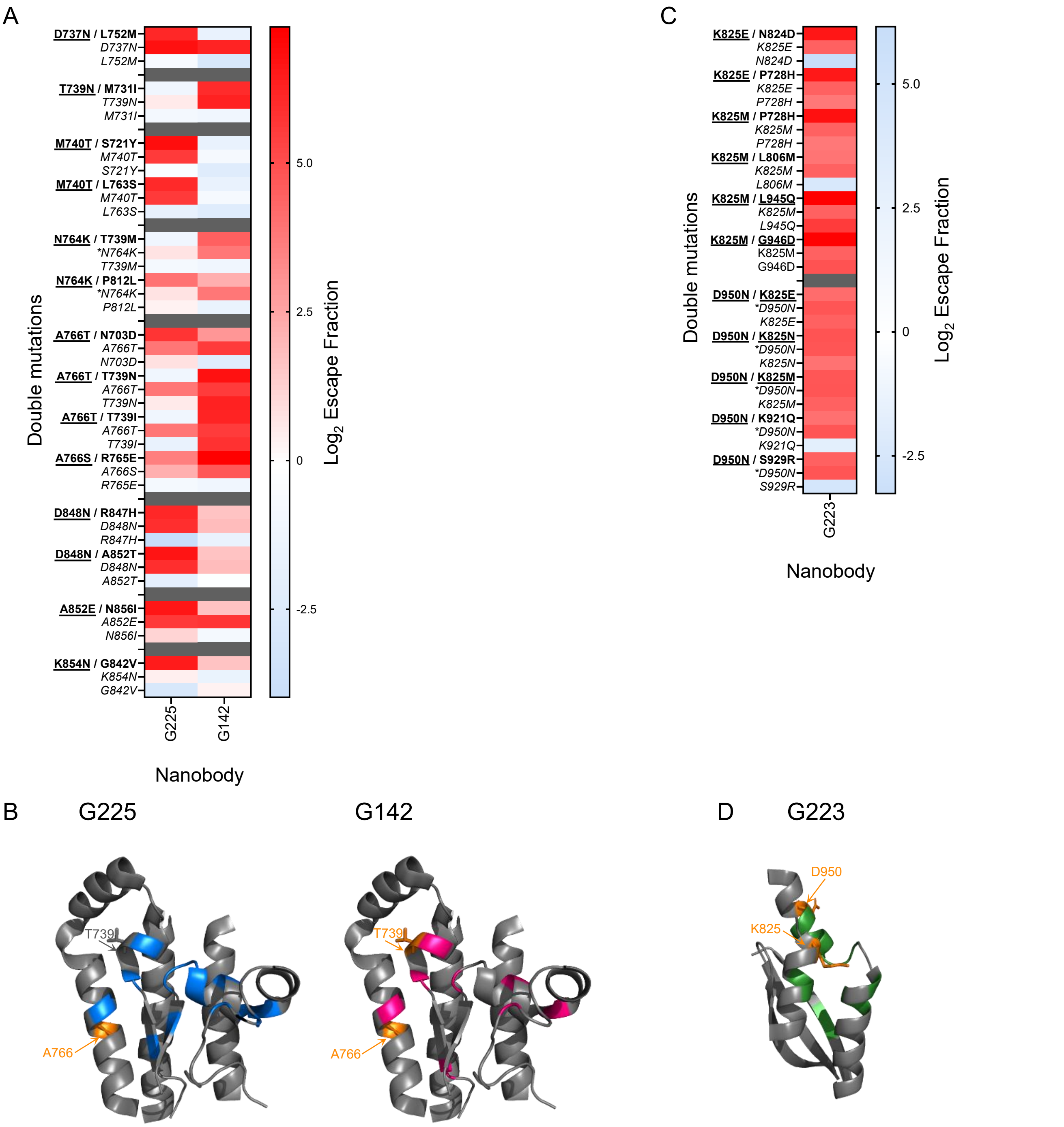

Supplementary Fig S5. Synergistic mutational combinations identified by yeast display deep mutational scanning. Extraction of escape fractions for those mutational combinations identified by deep mutational scanning for **(A)** G225/G142 and **(C)** G223. Only mutational combinations where at least one mutation was located near or within the epitope on the SARS-CoV-2 structure were analysed. Red represents mutation has a deleterious effect on binding whereas blue indicates a neutral effect. Escape fractions for each individual mutation is also shown and was extracted from the DMS of the single mutant population **(B)** G225 (blue) and G142 (pink) epitopes with synergistic

combination A766/T739 coloured in orange. **(D)** G223 epitope (green) with synergistic combination D950/K825 amino acids in orange. We estimate that combinatorial double mutant diversity is only a fraction (~0.37%) of the theoretical maximum diversity of over 100 million (528 residues in S2 gene $10{,}560^2$ double mutants). It is surprising that we were able to identify so many combinations of mutations that co-locate to the epitopes of G223, G225 and G142 given the limitations of library size of 3.8×10^6.
